# Genetic architecture of the tomato fruit lipidome

**DOI:** 10.1093/pnasnexus/pgaf401

**Published:** 2025-12-24

**Authors:** Anastasiya Kuhalskaya, Xiang Li, Jeongah Lee, Itay Gonda, Julia von Steimker, Mustafa Bulut, Esra Karakas, Josef Fisher, Konrad Krämer, Leah Rosental, Micha Wijesingha Ahchige, Karolina Garbowicz, Annabella Klemmer, Anne-Kathrin Ruß, Andreas Donath, Alvaro Cuadros-Inostroza, Wout Boerjan, Denise M Tieman, Dani Zamir, Harry J Klee, Saleh Alseekh

**Affiliations:** Max Planck Institute of Molecular Plant Physiology, Potsdam-Golm 14476, Germany; Department of Life Sciences, Ben Gurion University of the Negev, Beersheva 653, Israel; Anhui Province Key Laboratory of Horticultural Crop Quality Biology, School of Horticulture, Anhui Agricultural University, Hefei 230036, PR China; Horticultural Sciences, Genetics Institute, University of Florida, Gainesville, FL 32611-0690, USA; Max Planck Institute of Molecular Plant Physiology, Potsdam-Golm 14476, Germany; Unit of Aromatic and Medicinal Plants, Newe Ya’ar Research Center, Agricultural Research Organization, Ramat Yishay 30095, Israel; Max Planck Institute of Molecular Plant Physiology, Potsdam-Golm 14476, Germany; Max Planck Institute of Molecular Plant Physiology, Potsdam-Golm 14476, Germany; Program Center MetaCom, Leibniz Institute of Plant Biochemistry, Weinberg 3, Halle (Saale) 06120, Germany; Max Planck Institute of Molecular Plant Physiology, Potsdam-Golm 14476, Germany; Robert H. Smith Institute of Plant Sciences and Genetics, Faculty of Agriculture, Hebrew University of Jerusalem, Rehovot 7610001, Israel; Max Planck Institute of Molecular Plant Physiology, Potsdam-Golm 14476, Germany; Department of Life Sciences, Ben Gurion University of the Negev, Beersheva 653, Israel; Max Planck Institute of Molecular Plant Physiology, Potsdam-Golm 14476, Germany; Max Planck Institute of Molecular Plant Physiology, Potsdam-Golm 14476, Germany; Max Planck Institute of Molecular Plant Physiology, Potsdam-Golm 14476, Germany; Max Planck Institute of Molecular Plant Physiology, Potsdam-Golm 14476, Germany; Institute of Medical Informatics and Statistics, University Medical Center Schleswig-Holstein, Kiel University, Brunswiker Straße 10, Kiel 24113, Germany; Max Planck Institute of Molecular Plant Physiology, Potsdam-Golm 14476, Germany; MetaSysX GmbH, Potsdam-Golm 14476, Germany; Center for Plant Systems Biology, VIB (Flanders Institute for Biotechnology), Technologiepark 71, Ghent B-9052, Belgium; Department of Plant Biotechnology and Bioinformatics, Ghent University, Technologiepark 71, Ghent B-9052, Belgium; Horticultural Sciences, Genetics Institute, University of Florida, Gainesville, FL 32611-0690, USA; Robert H. Smith Institute of Plant Sciences and Genetics, Faculty of Agriculture, Hebrew University of Jerusalem, Rehovot 7610001, Israel; Horticultural Sciences, Genetics Institute, University of Florida, Gainesville, FL 32611-0690, USA; Max Planck Institute of Molecular Plant Physiology, Potsdam-Golm 14476, Germany; Center of Plant Systems Biology and Biotechnology, Plovdiv 4023, Bulgaria

**Keywords:** lipidomics, QTL, tomato, volatiles, lipoxygenase

## Abstract

The lipid composition of tomato (*Solanum lycopersicum* L.) fruit plays a crucial role in determining fruit quality, nutritional value, and the biosynthesis of key volatile organic compounds. Despite this importance, the metabolic diversity and genetic regulation of lipid composition in tomato fruit remain poorly understood. Here, we performed a genome-wide association study and QTL mapping for fruit lipid content from 550 tomato accessions and 107 backcross inbred lines in two consecutive seasons. Over 130 lipid compounds were identified in the population, allowing for the identification of over 600 metabolic QTL. We further described and validated candidate genes associated with lipid content. Among them is a lipase-like protein (*TomLLP*) whose function was validated in vivo using overexpression lines in tomato and knockout mutants in Arabidopsis. We also identified functions for three enzymes: a class III lipase (*Sl-LIP8*), a cyclopropane-fatty-acyl-phospholipid synthase (*CFAPS1*), and lipoxygenase C (*TomLoxC*). By utilizing knockout lines for *CFAPS1* and CRISPR-Cas9 loss-of-function lines for *Sl-LIP8* and *TomLoxC*, we demonstrated the functional importance of these enzymes in fruit lipid metabolism. Our study provides a comprehensive analysis of the tomato fruit lipidome and insights into key genes that shape natural variation in lipid content, establishing a framework for exploring how lipid dynamics may influence traits such as flavor and volatile formation.

Significance Statement Lipids are critical to fruit quality, yet their metabolic diversity and genetic regulation in tomatoes remain largely unexplored. Using genome-wide association study and QTL mapping, we dissected the tomato fruit lipidome and identified key genes driving natural variation. These findings provide a molecular foundation for understanding how lipid metabolism may influence fruit quality traits, including those associated with flavor and volatile composition.

## Introduction

Tomato (*Solanum lycopersicum L*.) is one of the most economically important crops in the world (1,), serving as an important source of diverse health-benefiting compounds, including vitamins, carotenoids, and phenolic compounds (2–5). In recent years, great progress has been made in understanding a wide range of metabolic traits associated with fruit compositional quality (6). Lipids in plants have essential structural roles within cells, as they are major constituents of membranes and constitute much of the cuticle layer that protects plant outer surfaces (7–9). In addition, lipids act as signaling molecules. One of the main defense hormones, jasmonic acid, is derived from linolenic acid (10), and fatty acids have been shown to directly induce the expression of defense-related R genes (11). Moreover, lipids serve as precursors for many compounds that contribute to flavor, that regulates attraction, and repulsion of herbivores and ultimately, seed distribution (12–15). In addition, so-called fatty acid–derived volatile organic compounds (FA-VOCs) are important contributors to human liking of food crops (16–18). In tomato, several FA-VOCs are significantly linked with overall liking and flavor intensity (12, 16, 19). Knowledge of how these volatiles are synthesized and how the pathways are regulated is important for the development of varieties with superior flavor that do not compromise plant defense.

Genetic mapping has been used to characterize and clone a large number of qualitative and quantitative traits in tomatoes, including pathogen resistance (20), fruit ripening (21), β-carotene formation (22), fruit morphology, and size (23–26). Genetic mapping of metabolite abundance enables the identification of metabolite QTL and potentially provides insights into the complex mechanisms underlying the regulation of metabolic pathways (12, 13, 27–35). Advances in metabolomic profiling coupled with an increase in genomic resources have enabled the identification of numerous metabolic QTLs (mQTLs) in tomato, for both primary (36–41) and secondary metabolites (29, 42–49). However, given the relatively low resolution reached using this approach, cloning of the causal genes can be challenging. Genome-wide association studies (GWAS) provide better QTL resolution (50, 51). Successful identification of complex traits in various crop species has been achieved by combining linkage QTL mapping using biparental populations and GWAS, which helps overcome the limitations of each approach (13, 32, 52). In tomatoes, several GWAS have been conducted, leading to, among other insights, an understanding of the history of tomato breeding and domestication. Examples include the 100-fold increased size of the modern tomato relative to its ancestor (53) as well as identification of QTL controlling morphological traits (54), volatile compounds (16, 19), and fruit metabolites (55–57). Nevertheless, utilization of the above approaches to investigate natural variation in the tomato fruit lipidome has not been thoroughly investigated.

Here, we describe large-scale lipid profiling of fruit pericarp tissue extracts of a GWAS panel as well as a *Solanum neorickii* backcross inbred line (BIL) population. We identified 436 and 175 mQTL using GWAS and linkage mapping, respectively. We identified 384 candidate genes associated with lipid content in fruit. To gain deeper insights into lipid metabolism in tomato fruit and any potential relationship to volatile compounds, we selected five genes for molecular characterization, namely acetyl-coenzyme A synthetase (*Solyc06g008920*), *Sl-LIP8* (*Solyc09g091050*) encoding a class III lipase, *CFAPS1* (*Solyc09g090510*) encoding a putative cyclopropane-fatty-acyl-phospholipid synthase, a lipase-like protein (*TomLLP, Solyc03g119980*), and *TomLoxC* (*Solyc01g006450*), encoding lipoxygenase C. To do so, we created and analyzed CRISPR-Cas9 knockout (KO) lines for *CFAPS1*, *Sl-LIP8*, and *TomLoxC*, and overexpression lines of *TomLLP*. In addition, we analyzed mutant lines of the *TomLoxC* Arabidopsis orthologue (*CSE, At1g52760*).

## Results

### A genome-wide lipidomic profile for tomato fruit

**Fig. 1.**
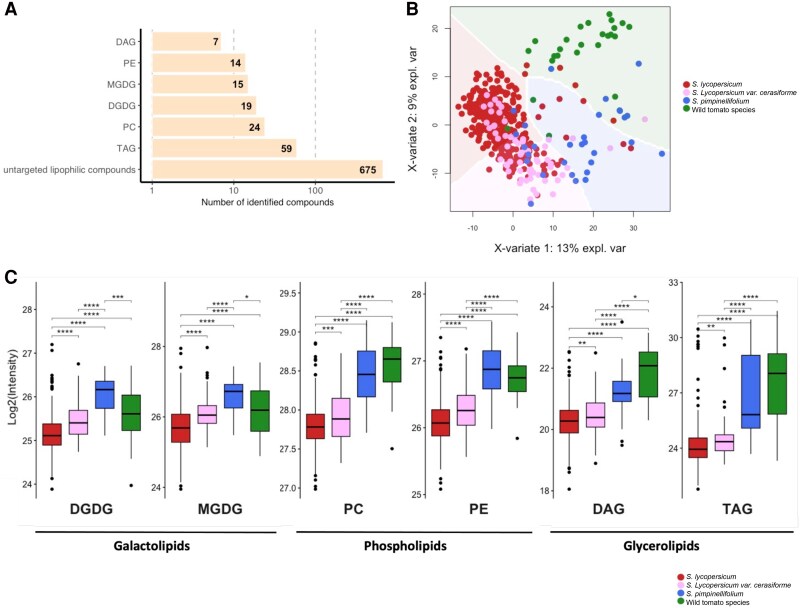
Characterization of natural variation in lipophilic metabolites across 550 different tomato accessions. A) Numbers of lipid compounds measured by LC–MS in 550 tomato accessions and their compound classes. B) PCA of lipid content for tomato lines representing green-fruited wild species, cultivated varieties, cherry tomato varieties *S. lycopersicum* var. *cerasiforme*, and red-fruited wild accessions of *S. pimpinellifolium*. Each dot represents a single accession. C) Box plots indicating the average value of all compounds for each lipid class in diverse wild accessions (*n* = 29), *S. pimpinellifolium* (*n* = 30), *S. lycopersicum* var. *cerasiforme* (*n* = 62), and *S. lycopersicum* (*n* = 398). Significances are indicated by *<0.05, **<0.01, ***<0.001 using Student's t test.

To evaluate the genetic underpinnings of the tomato fruit lipidome, we performed family-based QTL mapping using 107 BILs as well as GWAS on 550 tomato accessions grown in two harvest seasons (Fig. S1, Datasets S1–S6). Using high-throughput ultra-high performance liquid chromatography-mass spectrometry (UHPLC-MS), we were able to detect and quantify in total 138 lipid compounds (Fig. 1A and S2–S4, Dataset S5). Across two consecutive seasons, we identified 101 and 105 lipid species, respectively, with 72 of these species consistently identified in both harvests (Dataset S5). Quantified lipid compounds were classified into six major classes: seven diacylglycerols (DAGs), 19 digalactosyldiacylglycerols (DGDGs), 15 monogalactosyldiacylglycerols (MGDGs), 24 phosphatidylcholines (PCs), 14 phosphatidylethanolamines (PEs), and 59 triacylglycerols (TAGs). We also quantified 2,179 distinct lipophilic compounds from two populations (Datasets S1–S5).

We next performed a principal component analysis (PCA) of all annotated lipid compounds on the GWAS panel as well as the wild tomato relative, *Solanum pimpinellifolium* and other wild tomato species. This analysis revealed two main groups, consistent with the evolutionary and domestication relationship; tomato wild species including *S. pimpinellifolium*, and domesticated red-fruited accessions largely overlapped with *S. lycopersicum* var. *cerasiforme* (PCA, Fig. 1B). The abundance of different lipid classes was variable across the three groups (Fig. 1C). The wild tomato species had higher lipid levels compared with cultivated varieties, including glycero-, galacto-, and phospholipids. Moreover, most detected nonannotated lipophilic compounds were more abundant in older tomato varieties compared with domesticated ones (Fig. 1B and C). In addition to exploring the lipidome profiles in the GWAS panel, we quantified 83 lipids, and 826 distinct lipophilic compounds in fruits from a 107-member *S. neorickii* BIL (Fig. S4, Datasets S3 and S4). As expected, the lipid profiles exhibited a large variance across the *S. neorickii* lines.

### Genetic foundation of the tomato fruit lipidome

In order to uncover the genetic components of lipid abundances in fruit, GWAS was conducted in two consecutive seasons. We mapped the abundance of 134 annotated and 675 unidentified lipophilic compounds using 1.8 million single nucleotide polymorphisms (SNPs) (16). In addition, we used 16,526 SNPs generated by genotype by sequencing (GBS) analysis (58) on the GWAS population. We performed QTL mapping on 83 annotated and 826 unidentified lipophilic compounds from BILs using the 10K SolCAP single nucleotide polymorphism chip for linkage mapping.

**Fig. 2.**
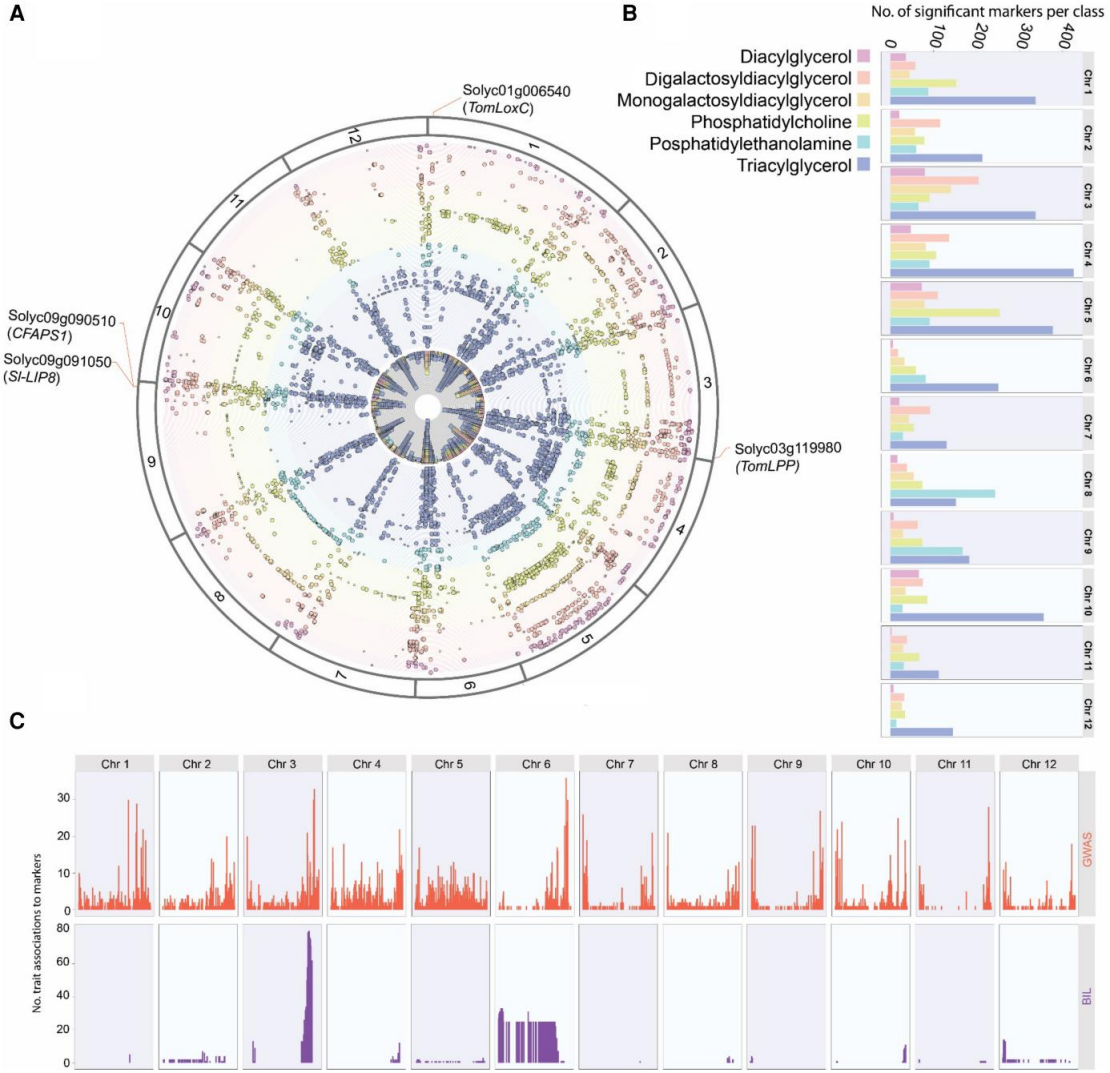
Pleiotropic map summarizing quantitative fruit mapping. A) Chromosomal distribution of the QTL derived from GWAS represents the combined results from the 2014 and 2015 seasons using SNPs markers generated from GBS and WGS. Colors indicate different lipid classes. The inner circle specifies the amount of lipids mapped to the identified region. QTL harboring candidate genes are highlighted. B) Bar charts show the number of significant SNPs associated with each lipid class chromosomewise. C) Number of traits associated with significant markers for GWAS on each chromosome (upper panel) and BIL (lower panel). The corresponding lipid compounds and number of QTL are provided in Datasets S1–S5.

The GWAS identified 436 significant associations indicating mQTL (*P* ≤ 1.0E−04; Dataset S6A). Additionally, linkage mapping using the *S. neorickii* BILs identified 175 more significant associations, representing mQTL in both homozygous and heterozygous lines (*P* ≤ 0.05; Dataset S6B and C). Visualizing the distribution of the mQTL in both (GWAS and BILs) populations indicated several hotspots for the regulation of a large number of lipids and lipophilic compounds in the tomato genome, particularly on chromosomes 1, 2, 3, and 6; these represent 12.8, 15.5, 11.4, and 17.3% of the detected loci, respectively (Fig. 2A, inner circle; Fig. S5, Dataset S6D). The vast majority of detected mQTL are for nonannotated lipophilic compounds followed by glycero- and galactolipids (Fig. 2B). Taken together, we were able to identify 34 conserved lipid mQTL across the tomato genome combining both GWAS and linkage mapping approaches (Fig. 2C, Dataset S6E).

### Key genes controlling lipid metabolism in tomato fruit

Having identified mQTL associated with lipid composition in fruit, we investigated several potentially causative genes. Among the identified loci, 384 candidate genes were predicted, based on sequence homology to genes that participate in fatty acid and lipid metabolism (Dataset S6). For example, phospholipase D (*Solyc04g082000*), is a potential causal gene for the observed changes in the level of phospho-, glycero-, and galactolipids in the QTL on chromosome 4 detected by GWAS in two consecutive seasons. Furthermore, an mQTL located on chromosome 3 identified through both GWAS and BIL mapping, contains a gene annotated as a class III TAG lipase, *Solyc03g123750 (SlLIP2)*. Another gene, *Solyc06g008920*, mapped only in the BIL population, affecting a wide range of long-chain saturated and unsaturated fatty acids, encodes an acyl-CoA synthetase/adenosine monophosphate (AMP) acid ligase II.

We focused further analysis on lipid-related genes potentially involved in the metabolism of lipid-derived volatiles, including *Sl-LIP8*, *TomLoxC*, and *CFAPS1*. Some of these genes, such as *Sl-LIP8*, have been previously implicated in the metabolism of fatty acid–derived volatiles (FA-VOCs) (12, 13, 57, 59). Here, we describe a role for *Sl-LIP8* in lipid metabolism, providing a link between lipid metabolism and FA-VOC synthesis. *TomLoxC* has also been shown to contribute to FA-VOC biosynthesis (13, 60–63). Notably, *TomLoxC* is located in a genomic region of the tomato that has been subject to selection during domestication. *CFAPS1* emerged as another promising lipid-related candidate potentially bridging lipid metabolism and FA-VOC synthesis. *TomLLP* was also selected to explore genetic diversity present in the *S. neorickii* backcrossed inbred lines (BILs). To complement the GWAS which focused on wild and cultivated accessions of *S. lycopersicum*, we used the *S. neorickii* BIL population as a genetically distinct and underexplored resource. This population carries unique wild alleles that are largely missing from cultivated tomatoes, offering the potential to discover novel loci involved in lipid metabolism.

**Fig. 3.**
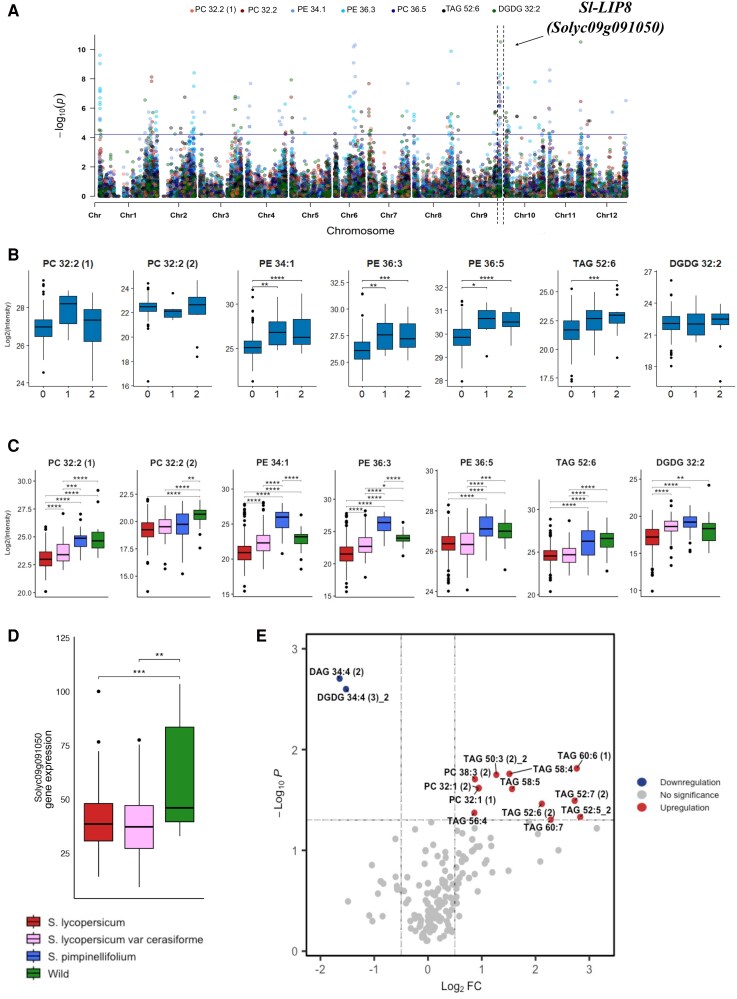
Lipid contents are associated with the locus harboring SI-LIP8 (Solyc09g091050). A) Manhattan plots of the mGWAS results using GBS SNPs data. B) Accessions were separated by the lead SNP and the average lipid level was determined. Zero represents the homozygous genotype for the common allele, one represents the heterozygote, and two represents the homozygous genotype for the other allele (C). The average lipid level in each of the following groups: *S. lycopersicum* (*n* = 398), *S. lycopersicum* var. *cerasiforme* (*n* = 62), *S. pimpinellifolium* (*n* = 30), and diverse wild tomato species (*n* = 27). D) SI-LIP8 transcript levels in fruits of *S. lycopersicum* (*n* = 258), *S. lycopersicum* var. cerasiforme (*n* = 56), and diverse wild tomato species (*n* = 6). E) Volcano plot showing the abundance of selected lipids in SI-LIP8 KO and wild type (Fla. 8059). Lipid levels were calculated as a log_2_-fold change of Fla. 8059. Significances are indicated by *<0.05, **<0.01, ***<0.001 using Student's t test.

An mQTL detected on chromosome 9 (Fig. 3A) contains an interval of about 50 genes, including *Sl-LIP8* (*Solyc09g091050*), which regulates the biosynthesis of short-chain FA-VOCs by cleaving 18:2 and 18:3 acyl groups from glycerolipids (57).

Next, using the lead SNP, the population was grouped according to SNP genotypes to identify accessions with allelic differences that could cause lipid level fluctuations and be potentially involved in the QTL variation (Fig. 3B). The process of domestication may affect these allelic differences. To examine this potential effect, we plotted lipid levels based on domestication status: starting with the wild ancestor, *S. pimpinellifolium*, progressing through *S. lycopersicum* var. *cerasiforme*, and ending with the domesticated *S. lycopersicum* species (Fig. 3C). Notably, wild tomatoes along with *S. pimpinellifolium* as well as accessions homozygous for the common allele showed significantly higher levels of all mapped lipids (Fig. 3B and C). Expression analysis using RNA-seq data from fruits of the entire GWAS population previously reported (56) showed that *Sl-LIP8* is highly expressed in wild species compared with *S. lycopersicum* var. *cerasiforme* and cultivated tomato (Fig. 3D).

In order to investigate the role of Sl-LIP8 in the tomato fruit metabolome, we generated *Sl-LIP8* CRISPR-Cas9 KO lines in the Fla. 8059 background and characterized their metabolites. Liquid chromatography (LC)–MS analysis of lipids in fully ripe fruit from KO lines and Fla. 8059 demonstrated notable alterations across various lipid classes demonstrating an example of inter-class-level regulation, where perturbation of a single gene affects different classes of lipid species (Dataset S7). The most substantial changes were observed in the contents of glycerolipids and phospholipids. Specifically, major differences were observed for polyunsaturated glycerolipids (Fig. 3E) confirming the influence of *Sl-LIP8* on lipid metabolism in tomato fruit.

**Fig. 4.**
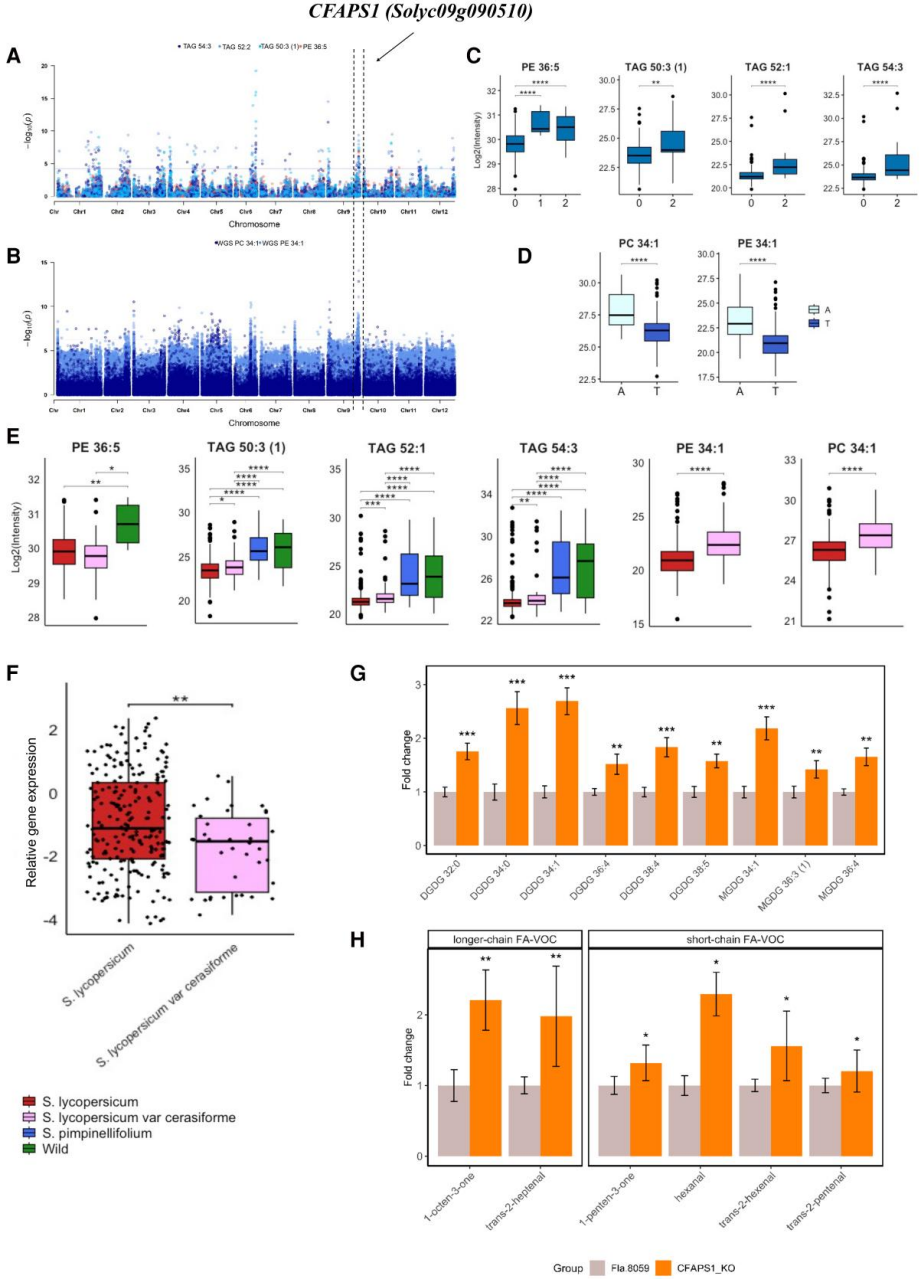
Phospho-, galacto-, and glycerolipid contents are associated with the CFAPS1 (Solyc09g090510) locus. A) Manhattan plots of mGWAS using GBS SNPs data. B) Manhattan plots of the mGWAS using WGS SNPs data. C) Lipid contents in accessions grouped by genotype classes at the lead mGWAS SNP. Zero is homozygous for the common allele; one is heterozygous; two is homozygous for the second allele. D) Lipid analysis of accessions separated by lead SNP alleles. E) The average lipid level in each of the following: *S. lycopersicum* (*n* = 398), *S. lycopersicum* var. *cerasiforme* (*n* = 62), *S. pimpinellifolium* (*n* = 30), and diverse wild tomato accessions (*n* = 27). F) CFAPS1 transcript level in fruits of *S. lycopersicum* (*n* = 240) and *S. lycopersicum* var. cerasiforme (*n* = 43). G) Lipidomic analysis of fully ripened CFAPS1 KO tomato fruits showing fold changes in specific galactolipids relative to control (Fla. 8059). Notably, DGDG 34:0 and DGDG 34:1 levels increased ∼2.5-fold, while MGDG 34:1 and DGDG 38:4 increased ∼2-fold in the KO lines. H) Abundance of FA-VOCs in CFAPS1 KO fruits relative to control. Short-chain volatiles (C5, C6), including hexanal, and longer-chain volatiles (C7, C8), such as 1-octene-3- one and E-2-heptenal, showed at least 2-fold increases in the KO lines. Significances are indicated by *<0.05, **<0.01, ***<0.001 using Student's t test.

We also detected another prominent mQTL located on chromosome 9 with a significant impact on the levels of phospho- and glycerolipids using both the GWAS mapping (Fig. 4A) and whole-genome sequencing (WGS) SNPs (Fig. 4B). The mQTL harbors two genes encoding putative cyclopropane-fatty-acyl-phospholipid synthases (Solyc09g090500 and Solyc09g090510). One of these genes, Solyc09g090510(CFAPS1), is expressed in developing fruits (https://bar.utoronto.ca/efp_tomato/cgi-bin/efpWeb.cgi). *CFAPS1* and *Sl-LIP8* seemed to locate close to each other; however, they are associated with different QTL (Dataset S6A).

Accessions responsible for the allelic variation were divided based on GBS (Fig. 4C) and WGS (Fig. 4D) lead SNPs, influencing lipid abundance. From wild tomato, domestication starts with *S. pimpinellifolium* (the closest known relative of modern tomatoes), continues with *S. lycopersicum* var. *cerasiforme*, and progresses to the domesticated *S. lycopersicum* species. Notably, the lipid levels, particularly TAG 54:3 and TAG 52:1 were significantly higher across *S. pimpinellifolium* and different wild tomato accessions (Fig. 4E) than in the cultivated the tomato group (Fig. 4E) as well as among accessions homozygous for the common allele (Fig. 4C), and accessions with lead SNP encodes for thymine (T) (Fig. 4D). The domestication process is likely responsible for these allelic variations.

RNA-Seq data obtained from fruits of the previously characterized GWAS population (56) showed that *CFAPS1 mRNA* was more abundant in cultivated tomatoes (Fig. 4F).

We created *CFAPS1* CRISPR-Cas9 KO lines and identified mutants with a deletion of 166 bp and an insertion of 19 bp in the promoter region and coding sequence (exon 1; Fig. S6), which resulted in a premature translation stop at the beginning of the protein. Lipidomic profiles in fully ripened fruit showed a 2.5-fold increase in DGDG 34:0 and DGDG 34:1 levels in the KO lines compared with the control (Fla. 8059). Other galactolipids, such as MGDG 34:1 and DGDG 38:4, also showed a 2-fold increase relative to the control (Fig. 4G, Dataset S8). In addition, we observed at least a 2-fold increase in the levels of short-chain (C5, C6) FA-VOCs, including hexanal, as well as at least a 2-fold increase in longer-chain (C7, C8) FA-VOCs, including 1-octene-3-one and *E*-2-heptenal (Fig. 4H, Dataset S8). Several of these volatiles are known to be derived from the breakdown of fatty acids, including those stored in glycerolipids and galactolipids (13, 60–63). The coincident increases in fatty acid pools and FA-VOCs are consistent with an interpretation where the fatty acid pools are limiting for flavor-associated VOCs and suggest that selection of genetic loci favoring higher levels of these precursors would improve fruit flavor.

**Fig. 5.**
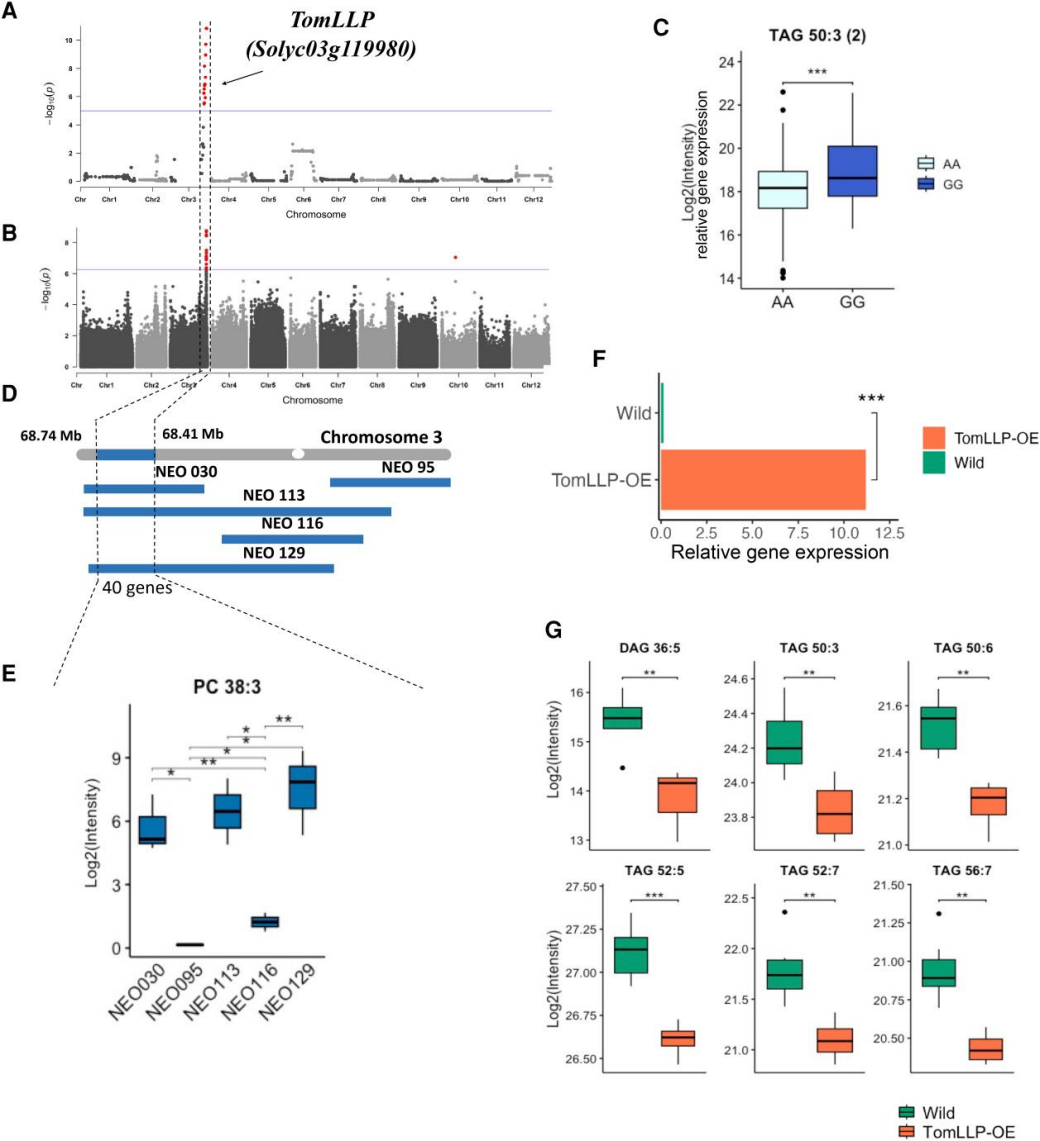
Linkage mapping identifies a role for TomLLP (Solyc03g119980) in fruit lipid metabolism. A) Association plot of PC 38:3 obtained with linkage mapping using *S. neorickii* BIL population. B) Manhattan plot of mGWAS of TAG 50:3 using WGS SNPs data. C) Lipid contents of accessions grouped by allelic classes defined by the lead mGWAS SNP. D) *S. neorickii* tomato segments introgressed into cultivated tomato variety TA209 on chromosome 3. E) Levels of PC 36:1 and PC 38:3 in BILs sharing the *S. neorickii* introgression on chromosome 3 and BILs with the TA209 background. F) TomLLP transcript levels in the TomLLP overexpression line and wild-type M82. G) Level of selected lipid in the TomLLP overexpression line and M82. Significances are indicated by *<0.05, **<0.01, ***<0.001 using Student's t test.

In addition to the above examples, both GWAS and linkage mapping identified a major mQTL influencing the levels of phospholipids and galactolipids at the end of chromosome 3 (Fig. 5A and B). Linkage disequilibrium (LD) analysis of GWAS and recombination breakpoints in a *S. neorickii* BIL population narrowed the mQTL region to 0.33 Mb. This region contains 40 genes, including *TomLLP* (*Solyc03g119980*), annotated as a lipase-like protein. The population was grouped according to SNP genotypes based on the lead SNP. These groups exhibited significantly different TAG 50:3 content (Fig. 5C). The *TomLLP* orthologue in *Arabidopsis thaliana*, *CSE* (*At1g52760*), encodes a caffeoyl shikimate esterase (CSE) that has been demonstrated to be involved in lignin biosynthesis (64). CSE has a dual enzymatic activity as both a monoacylglycerol acyltransferase and an acyl hydrolase (65, 66). TomLLP exhibits a strong phylogenetic relationship with CSE from other plant species (Fig. S7 and Dataset S9). Next, we selected five *S. neorickii* BILs covering the QTL interval (Fig. 5D and E) and measured transcript levels in the same fruit materials used to perform the lipid analysis. We detected higher expression of *TomLLP* in BILs harboring the *S. neorickii* allele compared with the BILs harboring the cultivated allele from the cv. TA209 (Fig. S8). To validate our finding, we generated a *TomLLP* overexpression (OE) line in the M82 tomato background carrying the *S. neorickii* allele driven by the figwort mosaic virus 35S promoter (Fig. 5F, Dataset S10). Lipid profiling of red ripe fruits from T2 plants showed significant differences in lipid contents between the overexpression line and wild type (Fig. 5G, Dataset S11) indicating a role for TomLLP in lipid metabolism and supporting its role as the causative gene associated with the QTL. Furthermore, leaf lipid profiling of three loss-of-function Arabidopsis *CSE* (*At1g52760*) lines demonstrated alterations in the level of multiple lipids belonging to six lipid classes (Fig. S9, Dataset S12). Taken together, the results support a role for *TomLLP* in tomato fruit lipid metabolism.

### Lipoxygenase, a key player affecting tomato fruit lipidome

**Fig. 6.**
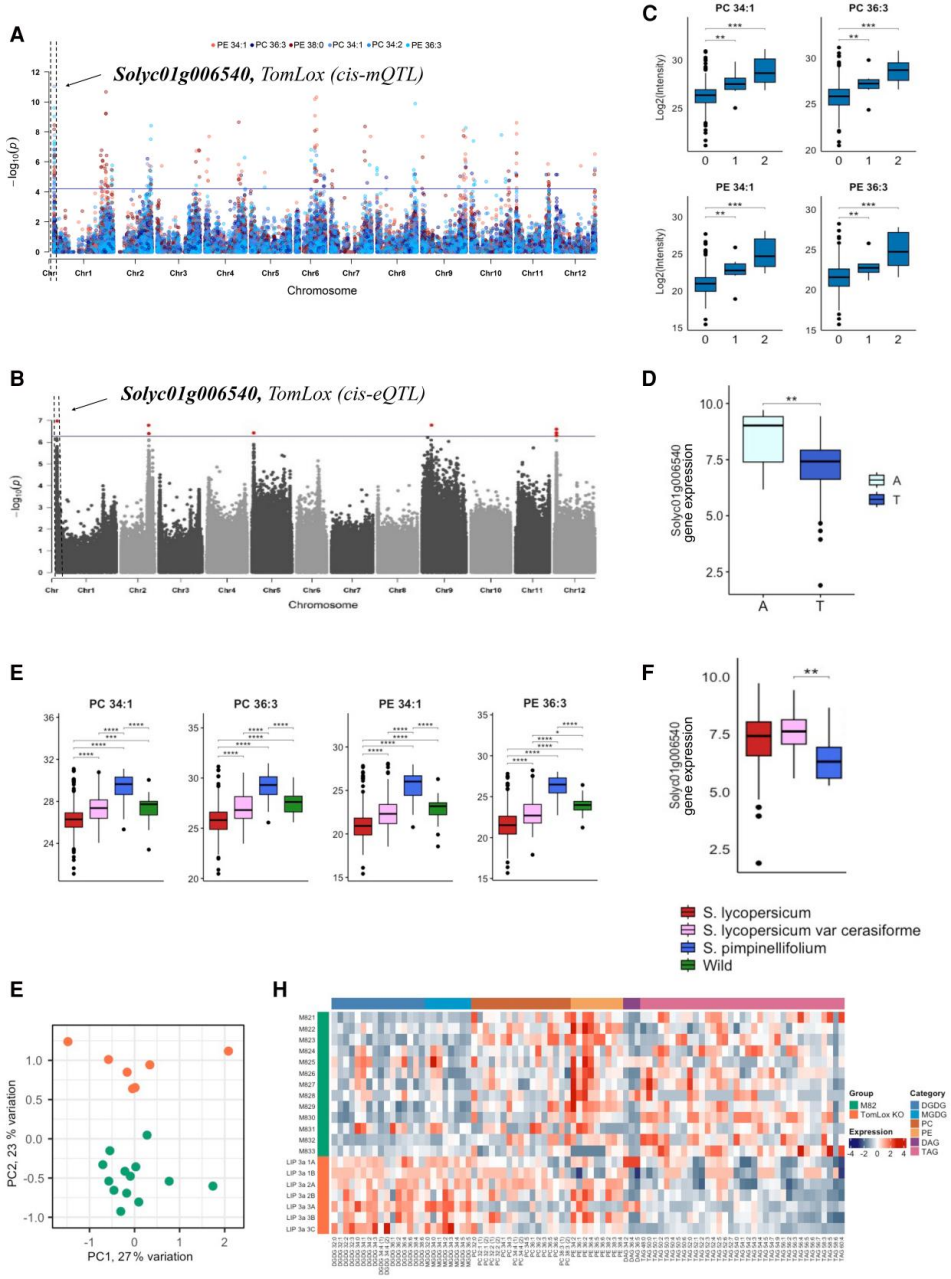
Phospho-, galacto-, and glycerolipid content is associated with the TomLoxC (Solyc01g006540) locus. A) Manhattan plots for mGWAS using GBS SNPs data. B) Manhattan plot for eGWAS using WGS SNPs data. C) Lipid contents for a group of accessions separated by the lead mGWAS SNP. Zero, homozygous for the common allele; one, heterozygous; two, homozygous for the second allele. D) Lipid contents for accessions grouped by the lead eGWAS SNP. E) Average lipid levels for *S. lycopersicum* (*n* = 398), *S. lycopersicum* var. *cerasiforme* (*n* = 62), *S. pimpinellifolium* (*n* = 30), and diverse wild tomato species (*n* = 27). F) TomLoxC transcript level in fruits of *S. lycopersicum* (*n* = 258), *S. lycopersicum* var. *cerasiforme* (*n* = 56), *S. pimpinellifolium* (*n* = 6). G) PCA plot of lipid levels in TomLoxC KO and wild-type M82. H) Heat map representing the abundance of short-chain FA-VOC (C5, C6) and longer-chain FA-VOC (C7, C8) in TomLoxC KO and wild type M82. Significances are indicated by *<0.05, **<0.01, ***<0.001 using Student's t test.

A robust association between lipids and the locus harboring *TomLoxC* (*Solyc01g006540*) was identified by GWAS (Fig. 6A). TomLoxC was previously shown to be involved in FA-VOCs production (12, 59). In order to identify expression differences across the GWAS population, we analyzed previously published *TomLoxC* expression data from 340 accessions (56) and performed eGWAS using 1.8 million SNPs data points (16). A *cis*-eQTL was detected for *TomLoxC* (Fig. 6B) indicating that this gene likely underlies the variation observed in the lipid levels mapped to the *TomLoxC* locus.

Accessions responsible for driving the allelic variation were identified based on the GBS lead SNP (Fig. 6C) affecting changes in lipid levels, and mapped variation in the *TomLoxC* transcript level based on the WGS lead SNP (Fig. 6D). When plotting the median expression level and lipid level associated with the lead SNP, we observed that low gene expression coincides with a high level of lipids (Fig. 6E and F). This pattern appears to reflect variation that may have been shaped during the evolutionary transition from wild to domesticated tomato. Specifically, several lipids associated with the *TomLoxC* locus showed higher abundance in *S. pimpinellifolium* compared with cultivated accessions, consistent with the gene expression data across the mapping panel (Fig. 6E).

To provide further insight into the role of *TomLoxC* in lipid metabolism and biologically validate our results, we generated *TomLoxC* CRISPR-Cas9 KO lines in the M82 background. Lipidomic profiling of red ripe fruit from KO lines and M82 revealed significant changes in a wide range of lipid classes (Fig. 6G and H, Dataset S13). The largest changes were observed in galactolipids followed by phospho- and glycerolipids. The levels of several polyunsaturated galactolipids including DGDG 32:3, DGDG 34:3, DGDG 36:6, MGDG 34:2, MGDG 34:5, and MGDG 36:5 were significantly lower in the control compared with the *TomLoxC* KO lines (Fig. 6H, Dataset S13). For instance, the level of DGDG 32:2 was 2.2 times higher in the TomLoxC KO lines than in the MM control. Similarly, DGDG 32:3 and DGDG 36:6 were 2.59 and 1.66 times higher in the KO lines, respectively. In addition, the levels of MGDG 34:2, MGDG 34:5, and MGDG 36:5 were increased by 1.68-fold, 2.78-fold, and 1.55-fold, respectively, in the *TomLoxC* KO lines compared with MM control. These results show a clear up-regulation of galactolipid levels when TomLoxC is disrupted, providing further support for its important role in regulating fruit lipid abundance.

We have previously shown that *TomLoxC* RNAi KO lines have greatly reduced contents of C5 and C6 FA-VOCs (59) and further identified a QTL associated with C5 (*E*-2-pentenal) and C6 (hexanal and hexanol) FA-VOCs (16). Taken together with the current data, we conclude that TomLoxC is a critical node in the flux from fatty acid precursors to multiple FA-VOCs that are essential for consumer liking of fruits and again, presents an important opportunity for allele selection in flavor improvement.

## Discussion

In recent years, the application of many metabolic GWAS studies has focused on dissecting the genetic architecture underlying regulation and biosynthesis of metabolic pathways (67). However, this approach has not yet been applied to investigate the fruit lipidome of tomato while also examining how lipid metabolism relates to fatty acid–derived volatile compounds and fruit flavor. Here, through a combination of GWAS and linkage analysis, we identified over 600 mQTL and many genes that affect lipid composition in tomato fruit. In order to validate the results, we determined the functions of four lipid biosynthesis genes by transgenic analysis.

Metabolite GWAS has become increasingly common in recent years, particularly for lipids (34, 35, 68). These studies have revealed novel associations between structural genes and lipids. In tomato, limited work based on the natural variation in a bi-parental population has been carried out to study cuticle lipids (7, 9). Here, we utilized both *S. neorickii* BILs and a GWAS panel to map the lipidome of tomato fruit pericarp. We examined the variation in lipid levels among cultivated and wild species (Datasets S1–S4). Wild tomato accessions had higher lipid levels as compared with cultivated tomatoes (Fig. 1C). Domestication of tomatoes has resulted in a narrower genetic and phenotypic variation in cultivated species (69–71 ). The utility of exotic germplasm as a source of new traits has been extensively exploited (27, 72–76). For example, introgression populations containing portions of the genomes of the wild tomato relatives *S. pennellii* and *S. neorickii* into cultivated tomato have provided useful tools to explore multiple genes with roles in morphological and metabolic traits (29, 38, 77, 78), including lipids (13). We selected the *S. neorickii* BIL population, which offers a well-structured yet underutilized resource that has not previously been profiled for lipids. This population contains unique wild alleles largely absent from cultivated germplasm and the GWAS panel, providing an opportunity to uncover novel loci. Its proven utility in previous QTL studies (77) therefore makes it useful to use as a complement to the GWAS approach. The large differences in chemical composition between fruits of the different species mean that introgressions of defined segments from those wild relatives frequently result in major perturbations of metabolic pathways. Our integrative approach using two types of populations has been proven to be particularly useful, allowing us to cross-validate some of the identified QTL and identify unique associations to each population (Figs. 1 and 2). That said, we identified 436 mQTL and 175 mQTL using GWAS and linkage mapping (*S. neorickii* BILs) approaches, respectively. Thirty-four mQTL and 38 candidate lipid-related genes were common between the two mapping populations.

The mGWAS identified an association between the levels of multiple lipid classes and the genomic locus containing *Sl-LIP8* (Fig. 3A). Mutants of *Sl-LIP8* exhibited significantly increased levels of several TAGs (Fig. 3E). Previously, through experimental KO mutants, we showed that *Sl-LIP8* encodes a class III lipase that cleaves TAGs and DAGs, releasing free fatty acids that are further converted into FA-VOCs (57). Moreover, Garbowicz et al. (13). precisely mapped the mQTL containing *Sl-LIP8* to a shared region within *S. pennellii* introgression lines (IL 9-3, IL 9-3-1, and IL 9-3-2), where reduced levels of DAGs, DGDGs, MGDGs, and TAGs are supporting the role of this gene in the observed QTL results and the function in the biosynthesis of important flavor-associated FA-VOCs (57) (Fig. S10). Taken together, our findings indicate that glycerolipid turnover mediated by Sl-LIP8 is associated with the accumulation of several short-chain FA-VOCs.

Previous results indicated that the transcript level of *Sl-LIP8* is higher in the *S. lycopersicum* cv. M82 tomato variety than in *S. pennellii* (13), potentially due to the structural variation in the promoter regions (79). In our study, we observed increased *Sl-LIP8* expression in wild tomato species such as *Solanum habrochaites, Solanum arcanum, Solanum Chmielewskii,* and *Solanum peruvianum*, which are more closely related to *S. lycopersicum* on the domestication continuum than *S. pennellii* (53, 80, 81). Additionally, the elevated *Sl-LIP8* expression was correlated with various lipid alterations (Fig. 3C and D).

The GWAS mapping uncovered an mQTL harboring the *CFAPS1* gene. Utilizing experimental CRISPR/Cas9-gene edited CFAPS1 mutants, we showed the role of CFAPS1 for the observed changes in various lipid classes and FA-VOCs (Figs. 4A and 3B). *CFAPS1* exhibited higher transcript levels in cultivated tomato accessions, consistent with corresponding differences in lipid levels (Fig. 4E and F). The *CFAPS1* gene encodes a cyclopropane-fatty-acyl-phospholipid synthase, a gene previously undescribed in tomatoes. In bacteria, this enzyme incorporates cyclopropane rings into unsaturated phospholipid membranes (82), while in plants, it modifies both phospholipid and galactolipid membranes, leading to the production of cyclopropane fatty acids (CFAs) (83). CFAs are believed to contribute to stress responses by enhancing membrane adaptation to environmental conditions such as pH, salinity, drought, and high temperatures (84). The tomato fruit ripening process involves chloroplast-to-chromoplast transition, membrane restructuring, fruit softening, and metabolite accumulation, including sugars and volatiles (85). CFAs are synthesized on polar membrane lipids such as phospholipids and galactolipids and subsequently stored in neutral TAGs (86, 87). In plants, these TAGs may later serve as substrates for certain plant biosynthesis pathways such as specific FA-VOC production (13, 57) (Fig. S10). In particular, TAG 54:6 and TAG 54:9 are key intermediates in the lipid pathway, acting as precursors for fatty acid–derived volatiles such as C5 and C6 VOCs. These TAGs can be hydrolyzed by lipase family members to release FA 18:2 and FA 18:3, which are used for volatile synthesis (57).

We mapped phospholipid (e.g. PE 46:5) and glycerolipid (e.g. TAG 50:3, TAG 52:1, TAG 54:3) contents to the genomic region containing *CFAPS1*, suggesting that allelic variation at this locus could underlie the observed changes in lipid levels. Functional disruption of *CFAPS1* led to widespread alterations in galactolipid composition, including at least a 2-fold increased level of membrane DGDG and MGDG species (Fig. 4G, Dataset S8), highlighting its broader central regulatory role, affecting multiple lipid biosynthetic pathways. In plants, membrane lipids include both phospholipids and galactolipids (88). This broader lipidomic phenotype suggests that *CFAPS1* may act as a central regulatory node, influencing multiple lipid biosynthetic pathways. These findings also show a clear example of inter-class-level regulation, where perturbation of a single gene affects lipid species across distinct classes.

In parallel, *CFAPS1* KO lines showed substantial increases in short-chain (C5, C6) and longer-chain (C7, C8) FA-VOCs, including hexanal (C6), *E*-2-hexenal (C6), *E*-2-heptenal (C7), and 1-octen-3-one (C8) (Fig. 4H). In tomatoes, specific FA-VOCs, particularly C5, C6, C7, C8, and C10 compounds, are strongly associated with flavor intensity and consumer preference (16, 19). Our findings identify *CFAPS1* as a key gene underlying natural variation in lipid abundances and reveal its association with changes in volatile profiles. Therefore, during the chloroplast-to-chromoplast transition during fruit ripening, glycerolipids, phospholipids, and galactolipids may serve as precursors for FA-derived volatiles (Fig. S10). This insight provides a broader systems-level understanding of how lipid metabolism contributes to fruit quality traits, extending beyond membrane composition to include aroma and flavor profiles.

Another gene that was shown to participate in the synthesis of FA-VOCs, through both lipase-dependent and lipase-independent pathways, is *TomLoxC* (13, 60–63). Quantitative variation in several lipids was mapped to the *TomLoxC* locus (Fig. 6A). Further support for *TomLoxC* as the causal gene is provided by a *cis*-eQTL (Fig. 6B). *TomLoxC* has higher transcript abundance in cultivated varieties (Fig. 6F). Fruit ripening and senescence are accompanied by disorder of ROS metabolism, resulting in excessive accumulation of ROS, resulting in continuous lipid peroxidation and membrane injury. Earlier research documented that *TomLoxC* is a chloroplast-targeted lipoxygenase active during ripening of tomato fruit. *TomLoxC* utilizes both linoleic and linolenic acids as substrates, resulting in the production of flavor-associated short-chain FA-VOCs (12, 59). Lipid profiling of a *TomLoxC* loss-of-function line revealed that *TomLoxC* is associated with at least 2-fold increases in multiple lipid species with the most significant changes occurring in galactoplipids such as DGDG 38:6, DGDG 36:2, MGDG 34:4, MGDG 32:0, and others (Fig. 6H, Dataset S13). Thus, the association of various phospholipids with the *TomLoxC* gene and accumulation of galacto-, phospho-, and glycerolipids in the KO lines supports a role for this enzyme in chloroplast lipid degradation during fruit ripening concomitant with the release of free FAs that are the precursors for ripening-associated FA-VOCs (Fig. S10). Taken together, we validated a role for *TomLoxC* as a causal gene responsible for at least part of the natural variation of lipid as well as FA-VOC abundance in tomatoes and their close relatives.

Our results indicate that *Sl-LIP8, CFAPS1,* and *TomLoxC* form part of a network linking lipid metabolism with pathways associated with FA-VOC accumulation. Several of these FA-VOCs have been demonstrated to affect consumer preferences (14, 16, 18). The *TomLoxC* locus appears to be under positive selection within a domestication sweep (53). Thus, selection of appropriate alleles for all of these genes offers substantial opportunities for breeding tomato varieties with improved flavor.

We also identified a region on chromosome 3 from both mapping populations that contains *TomLLP* that affects the levels of phospholipids and galactolipids (Fig. 5A and B). *TomLLP* is annotated as a lipase-like protein and clusters closely with the CSEs found in *S. tuberosum* and *Capsicum annuum*, rather than with other tomato lipases (Fig. S7). The Arabidopsis orthologue (*At1g52760*), CSE participates in lignin and lipid biosynthesis (64) (Fig. S10). Additionally, prior research has demonstrated that *At1g52760* exhibits lysophospholipase activity, utilizing lysophospholipids as substrates, which has a role in phospholipid metabolism (65, 66, 89) and also has acyltransferase activities, facilitating the synthesis of DAG and TAG (66). Overexpressing *TomLLP* in tomatoes resulted in decreased glycerolipid contents (Fig. 5F and G), while lipid profiling of three Arabidopsis loss-of-function *cse* mutants (*At1g52760)* exhibited a significant increase of multiple lipid species that belong to six lipid classes (Fig. S9, Datasets S11–12). In some plants, stress and lipid peroxidation during postharvest storage are linked to lignin buildup (90) (Fig. S10). While the impact of lipid metabolism on fruit texture degradation and aging was studied (85), its connection to lignin accumulation remains unclear. Lignin formation starts in the phenylpropanoid pathway, producing monolignols, which then cross the cell membrane and polymerize in the cell wall. Loss of membrane integrity may lead to uncontrolled movement of monolignols and enzymes, boosting lignin production (Fig. S10). Most research has focused on the role of reactive oxygen species (ROS) in lignin-related enzymes, with little attention given to how lipid peroxidation and membrane damage contribute to lignin synthesis. Further study is needed to explore how lignin accumulation relates to energy metabolism and lipid peroxidation in postharvest fruits (90). Our results illustrate that both *TomLLP* and *CSE* function in lipid metabolism. Further study is needed to explore if *TomLLP* also has a dual function as does *CSE*.

This study combined lipidomics with mGWAS and family-based QTL mapping to identify novel lipid-metabolism genes and expand our understanding of genome-level regulation of lipid biosynthesis in tomato. Hence, those genes are likely to be useful in breeding for the improvement of palatability and nutrient contents.

## Materials and methods

### Plant material

The GWAS mapping population used in this study comprised a collection of 550 *S. lycopersicum* accessions that were selected after a phenotype-guided screen of over 7,900 tomato accessions from around the world (58). The GWAS panel included modern cultivars, heirloom strains including *S. lycopersicum* var*. cerasiformie*, plus wild tomato species (*S. habrochaites, S. arcanum, S. chmielewskii, S. peruvianum, S. neorickii, S. pinnellii, Solanum corneliomuelleri, Solanum huaylasense, Solanum galapagense, Solanum chilense*, and *S. cheesmaniae*) harvested from two greenhouse experiments in fall 2014 and fall 2015. Wild accessions were excluded for the fall 2014 and fall 2015 seasons. For the GWAS mapping experiment, we used accessions of S*. lycopersicum,* and *S. lycopersicum* var*. cerasiforme* (91, 92). Each season's GWAS mapping was done independently, with each accession used once. About 50% of the accessions overlapped between the two consecutive harvests. The fall 2015 experiment (season) comprised a collection containing 388 *S. lycopersicum* accessions (cultivated tomato), 61 accessions of the *S. lycopersicum* var. *cerasiforme,* 30 *S. pimpinellifolium* accessions, and 25 wild accessions. The 2014 season comprised a panel of 295 accessions including 24 *S. lycopersicum* var. *cerasiforme* cherry tomato accessions and 271 cultivated varieties.

The *S. neorickii* BILs resulted from a cross between the green-fruited, self-compatible wild accession LA2133 and the processing tomato inbred variety cv. TA209 (*S. lycopersicum*) and contained 142 lines, due to poor germination, only 107 lines were used for this study (Fig. S11) (77). Additionally, hybrids for all BILs were produced in the background of the cv. TA209 recurrent parent to evaluate the wild introgressions in a heterozygous state.

For both the GWAS panel and the *S. neorickii* BILs population, pericarp tissue was isolated from ripe fruits, snap frozen in liquid nitrogen and stored at −80 °C before extraction.

*Sl-LIP8*, *CFAPS1* KO lines and wild-type Fla.8059 were grown in randomized, replicated plots in a heated greenhouse on the University of Florida campus or a field in Live Oak, FL, using recommended commercial practices. All fruits for lipid and FA-VOC quantification were harvested at a full-red ripe stage.

### Lipid extraction and UPLC–FT–MS measurement

Lipids were extracted from the GWAS panel harvested in two consecutive years from plants grown in the greenhouse and two to four independent biological replicates of *S. neorickii* BILs from fruit pericarp (93 ). Briefly, 120 mg of ripe frozen fruits were used to make aliquots. Lipids were extracted with 1 mL of precooled (−20 °C) extraction buffer (homogenous methanol:methyl-*tert*-butyl-ether (1:3) mixture + internal standards). After 10 min incubation in 4 °C and sonication for 10 min in a sonic bath, 500 µL of water/methanol mixture was added. Samples were then centrifuged (5 min, 14,000 × *g*). The lipophilic phase was collected and dried under vacuum. Pooled samples were used as a quality control at the beginning, in the middle and in the end of each day of LC–MS run. Samples were processed using ultra-performance LC coupled with Fourier transform MS (UPLC–FT–MS) on a C_8_ reverse phase column (100 × 2.1 mm × 1.7 µm particle size, Waters) at 60 °C. Samples were first subjected to LC to separate the components. The mobile phase consisted of 1% 1 M NH_4_OAc and 0.1% acetic acid in water (buffer A) and acetonitrile/isopropanol (7:3, UPLC grade BioSolve) supplemented with 1 M NH_4_OAc, 0.1% acetic acid (buffer B). The dried lipid extracts were re-suspended in 500 µL buffer B. The following gradient profile was applied: 1 min 45% A, 3 min linear gradient from 45% A to 35% A, 8 min linear gradient from 25 to 11% A, 3 min linear gradient from 11 to 1% A. After washing the column for 3 min with 1% A the buffer was set back to 45% A, and the column was re-equilibrating for 4 min, leading to a total run time of 22 min. The flow rate of the mobile phase was 400 µL/min. Mass spectra were acquired using an Exactive mass spectrometer (ThermoFisher, http://www.thermofisher.com) equipped with an ESI interface.

### Targeted lipid profiling by LC–MS and data acquisition

Processing of chromatograms, peak detection, and integration were performed using REFINER MS 10.0 (GeneData, www.genedata.com). Obtained mass features characterized by specific peak ID, retention time, *m/z* values, and intensity were further processed using in-house R scripts (Team, 2000). Clusters with mean signal intensities lower than 40,000 were removed and only peaks present in at least 80% of the samples were kept for analysis. Peak intensities were weight- and day-normalized and log_2_-transformed. After that, obtained molecular features were queried against in-house database for annotation.

### VOC collection and analysis

The measurement of VOC was conducted following the method described by Li et al. (57). In brief, ripe fruits were collected from three to four weekly harvests, with each harvest consisting of six pooled fruits. Volatiles were extracted from 100 g of chopped fruits over a 1-h period. These volatiles were trapped on a divinylbenzene resin column (Super Q) and eluted with methylene chloride, using nonyl acetate as an internal standard. The samples were then separated on a DB-5 column (Agilent) and analyzed using an Agilent 6890 N gas chromatograph. Retention times were compared with known standards, and the identities of volatile peaks were confirmed by gas chromatography–MS (Agilent 5975, www.agilent.com).

### General statistical and multivariate analysis of lipidomic data

Principal component analysis plot, boxplot analysis, pleiotropic map, volcano plot, heat maps for the lipidomic data were obtained using R software version 4.3.1.

The chromosomal distribution of mQTL identified was obtained by applying the RIdeogram R package to visualize and map genome-wide data on idiograms using R software version 4.2.2 (94).

Heat maps (Figs. S2–S4) were created using Multi Experimental Viewer (MeV) software. Lipids were clustered using complete-linkage clustering.

### GWAS and linkage QTL mapping

Accessions for mGWAS were genotyped by sequencing (GBS) at Cornell University following an established protocol (95) which yielded 16,526 SNP markers (58) all marker data are deposited in the Phenome Networks database (http://unity.phenome-networks.com). The final SNP matrix (16,526) used for the analysis was obtained by filtering for minor allele frequency ≥5%. Three principal components were included in the mixed linear model (MLM) to account for population structure and the SNP fraction considered for PCA. The kinship matrix and other parameters were set to default values. Association analysis was conducted using both 16,526 SNPs obtained from the GBS on 550 accessions and 1.8 million SNPs across 367 overlapping (same) accessions were previously characterized (16). GWAS was performed using a compressed MLM (96) implemented in the Genome Association and Prediction Integrated Tool (GAPIT) in R (97).

The *S. neorickii* BILs were genotyped with a 10K SolCAP single nucleotide polymorphism chip, and 3,111 polymorphic markers were used for mapping using “qtl” package version 1.40-8 that follows Haley–Knott regression (77) (Fig. S11).

### QTL identification and candidate gene selection

We extracted SNPs associated with lipid species with significant *P-*values. For the mGWAS with GBS SNPs (*n* = 16,526) data, the *P*-value was calculated according to the formula <1/*n* (*n* = 16,526). For mGWAS with 1.8 million SNPs data, the *P*-value was calculated according to the formula <1/*n* (*n* = 1,800,000) (32).

Candidate loci in the GWAS were identified based on the logarithm (base 10) of odds (LOD) score. A LOD score ≥4.5 was chosen as significant for mGWAS using GBS SNP data. A LOD score ≥6.5 was chosen as significant for mGWAS using 1.8 million SNP data. All genes in a given QTL were taken as putative candidates. Candidate genes were selected for validation based on their sequence homology with Arabidopsis genes related to lipid metabolism, their tissue-specific expression, and functional annotation.

The lead SNPs are defined as SNPs with the most significant *P*-value and with the highest LOD score. The other SNPs in this region may only have an association because they are in LD with the causal SNP or they may be independently associated.

The lipid profile of each *S. neorickii* BIL was compared (i.e. ANOVA, using permissive threshold, *P* < 0.05) to the lipid content of TA209. If it was significantly different from the TA209 genotype, the introgression was considered as harboring an mQTL. Causal genes responsible for mQTL were identified considering the margins of introgressed regions from *S. neorickii* delimited by the genetic markers used in this work. The upstream and downstream borders of each introgression were established to be halfway between the inclusive and exclusive wild-species SNP (77).

### LD and allelic class analysis

Allelic class analysis was conducted using available SNP data. Accessions were grouped according to shared SNP genotypes at the lead locus, and the median lipid value was calculated for each group. To explore patterns of allelic similarity, accessions were clustered based on allele-sharing distance using Ward's minimum variance method (98). One-way ANOVA followed by multiple comparison testing (*P* < 0.05) was used to identify lipid features significantly differing across the SNP-defined allelic groups.

### Cloning of lipase-like protein (*TomLLP*) and generation of transgenic plants

Gateway Technology (Invitrogen) was used in this work for overexpression of the lipase-like protein (*Solyc03g119980*) under the control of the Cauliflower mosaic virus *35S* (*CaMV35S*) promotor. Agrobacterium-mediated transformation of *S. lycopersicum* cv. M82 was performed following the protocol previously established in our institution. This M82 background was chosen due to its high transformation efficiency compared with wild species such as *S. neorickii*, facilitating efficient generation of transgenic lines (99). The pDONR221 vector (4761 bp) for *attB-attP* reaction with a selectable marker for kanamycin resistance was used. The pK7WG2 (11,159 bp) vector for overexpression contained the *Solyc03g119980* gene in the sense orientation under the control of the *CaMV35S* promoter. Transgenic plants were selected on kanamycin-containing media, and successful integration and expression of the transgene were confirmed by PCR and quantitative RT-PCR, respectively.

### qPCR gene expression analysis

Three parental M82 and six independent transgenic lines (with overexpressed *Solyc03g119980*) were grown in a greenhouse (Dataset S13). The plants were grown in pots with 20 cm diameter 3 L pots. At least three biological replicates were grown for each genotype. At least six fruit samples were collected from each replicate for transgenic lines and ten samples for the parental M82 line. Fruit pericarp was frozen in liquid nitrogen before storage at −80 °C. Frozen fruit pericarp was ground and RNA was extracted using a Thermo Fisher Scientific kit. The primers for *Solyc03g119980* were designed based on the Primer3 tool. The housekeeping genes were chosen according to the relevant literature (Dataset S14) (100). The differences in gene expression were calculated as fold change between independent transgenic lines and M82 according to the method of Schmittgen and Livak (101) Δ*C_t_* value was calculated as the difference between the *C_t_* (cycle threshold) of the candidate gene and the *C_t_* of the control gene for normalization of gene expression, according to Schmittgen and Livak (101).

### CRISPR lines

Three CRISPR lines for *TomLoxC* (*Solyc01g06540*) were created following the protocol described by Reem and Van Eck (102) with minor modifications. CRISPR constructs were created with a two-step golden gate cloning procedure to assemble a vector containing two guide-RNA-expressing cassettes, a kanamycin resistance gene, and the Cas9 nuclease, which was introduced into *Agrobacterium tumefaciens* GV2260. Guide RNAs were designed using the online tool CRISPR-P 2.0 (103). Transgenic plants were produced as previously described by Reem and Van Eck (102). Cas9-free plants homozygous for the gene of interest were transplanted in the greenhouse.

The design procedure and efficacy test of sgRNAs were performed using the CRISPR-P (http://cbi.hzau.edu.cn/cgi-bin/CRISPR) tool and the Guide-it sgRNA In vitro Transcription and Screening System (Takara, Mountain View, CA, United States) according to the manufacturer. Vector construction and tomato transformation were performed as described previously (57). Briefly, two 20-bp sgRNAs were inserted into a CRISPR/Cas9 binary vector (pCAMBIA2300_CR3-EF), in which the target sequence was driven by the Arabidopsis *U6-26* promoter and *Cas9* by 2 × 35S. The sgRNA sequences are listed in Dataset S14. The final binary vector was transformed into cultivar Fla. 8059 by *Agrobacterium*-mediated transformation (57). Genomic DNA was extracted from T1 and T3 homozygous *cfaps1* leaves and flanking regions containing the target sites were amplified using the specific primers *CFAPS1*-F and *CFAPS1*-R. The homozygous *cfaps1* allele was verified in the T1 and T3 generation by PCR-based sequencing. Cas9-free plants were used for quantitative analysis. The primers used for amplification and genotyping are listed in Dataset S14.

Plant material from CRISPR lines targeting SI-LIP8 (*Solyc09g091050*) have been previously published (57). The sgRNAs were inserted into the pCAMBIA2300_CR3-EF vector and transformed into Fla. 8059 by *Agrobacterium*-mediated transformation. Genomic DNA from an F2 plant backcrossed to WT and T3 tomato leaves was used for amplification with specific primers (Sl-LIP8-F and Sl-LIP8-R) for genotyping. Quantitative analysis used Cas9-free plants (57).

To generate *CFAPS1* KO mutants, we designed the sgRNA close to the 5′ end of the gene to maximize the likelihood of disrupting gene function. This approach resulted in a large deletion spanning both the promoter region and the coding sequence (exon 1), along with a 19-bp insertion and a 166-bp deletion. The mutation caused a premature translation stop near the beginning of the protein, effectively eliminating *CFAPS1* function (Fig. S6).

### Experimental design for TomLLP (*Solyc03g119980*) validation

Sterilized seeds were grown on Murashige and Skoog selective plates with 20% sucrose (2MS) and Km under long-day conditions (16 h light, 8 h dark), temperature was kept at 21/16 °C (day and night, respectively), light intensity at 150 μE m^−2^ s^−1^, humidity 75%. After 3 weeks, seedlings that survived selection were transported to the greenhouse in individual round pots with soil (potting compost) for fruit production and seeds for the next generation. The plants with empty-vector control and wild-type plants were grown in the same conditions as plants with overexpressed candidate genes.

### Experimental design of the Arabidopsis orthologue (*CSE, At1g52760*) of candidate gene (*TomLLP*, *Solyc03g119980*) validation

AtCSE_KD (*cse-1*, SALK_008202C) and AtCSE_KO1 (*cse-2*, SALK_023077) have been previously described (64, 65). AtCSE_KO2 (GABI_368D11) is a T-DNA insertion mutant and was obtained from the GABI-Kat collection (104). The T-DNA flanking sequence was analyzed via PCR with the primers 5′-ACCATTAGATGGTGAAATCAAAGG-3′ (1) and 5′-ATAATAACGCTGCGGACATCTACA-3′ (2), whereas the absence of the T-DNA was analyzed via PCR with the primers 1 and 5′-CTTGATAGCCTTCCCAACCA-3′ (3). The AtCSE_KO2 T-DNA insertion was confirmed to be positioned in the second exon (64).

## Supplementary Material

pgaf401_Supplementary_Data

## Data Availability

The authors declare that the data supporting the findings of this study are available within the paper and its supplementary information files.

## Preprints

This manuscript was posted on a preprint: https://doi.org/10.1101/2024.07.08.602461.
