# Supplementary material for: Genetic architecture of the tomato fruit lipidome
Source: PNAS Nexus. 2025 Dec 24;5(1):pgaf401. doi: 10.1093/pnasnexus/pgaf401 (PMC12781095; doi:10.1093/pnasnexus/pgaf401)
Supplement: pgaf401_Supplementary_Data [file pgaf401_supplementary_data.zip › PNASNEXUS-PNASNEXUS-2024-01465RR-s02.pdf]

Genetic architecture of the tomato fruit lipidome

Supplemental Figures S1-11

Figure S1

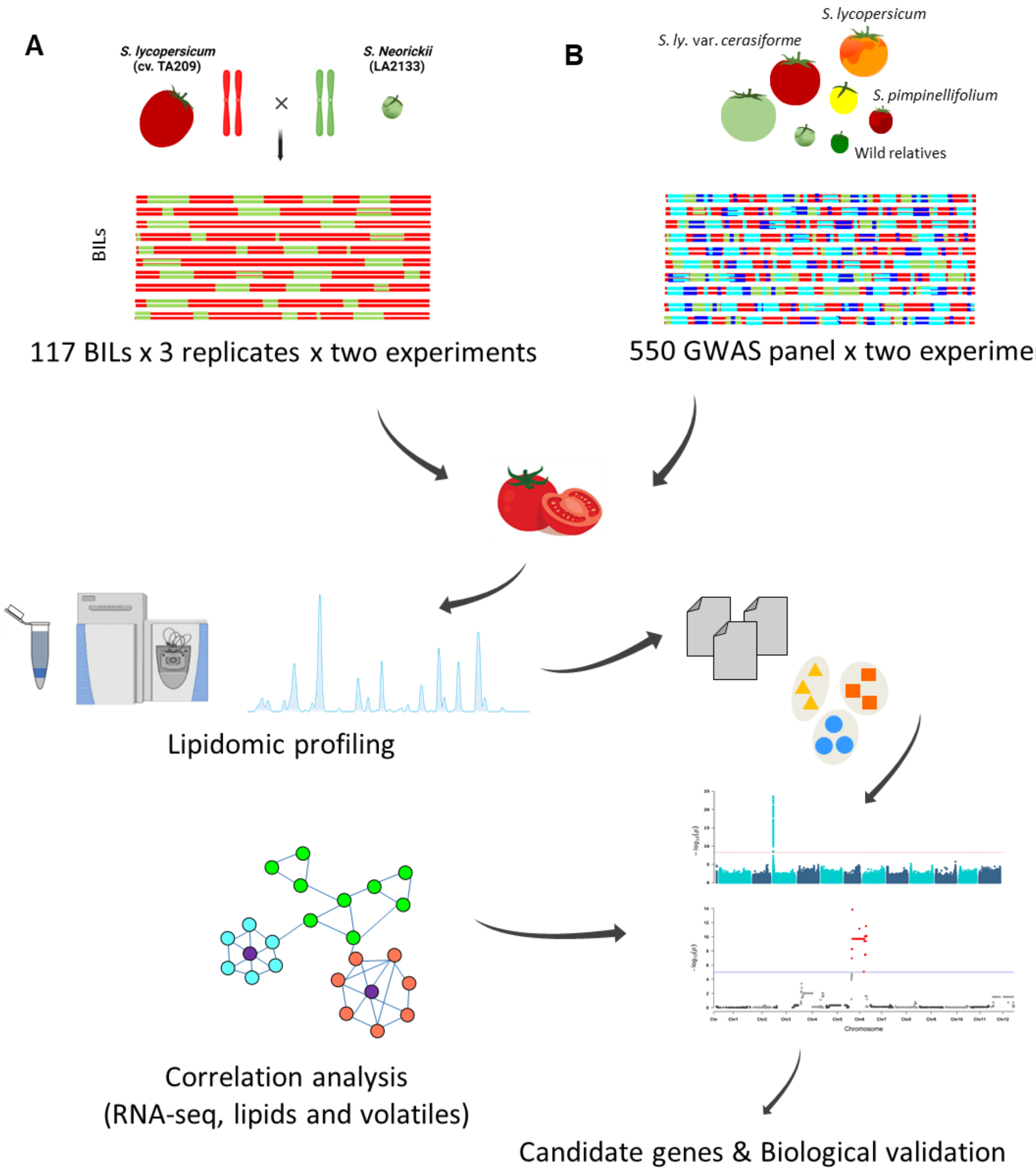

Figure S1. Schematic model of conducted experiments focused on investigation of genes underlying lipid metabolism in tomato fruit pericarp applying forward genetic approaches using association panels represented by (A) *S. neorickii* biparental population, and (B) unrelated cultivated tomato genotypes for genome-wide association study (GWAS).

Figure S2

A

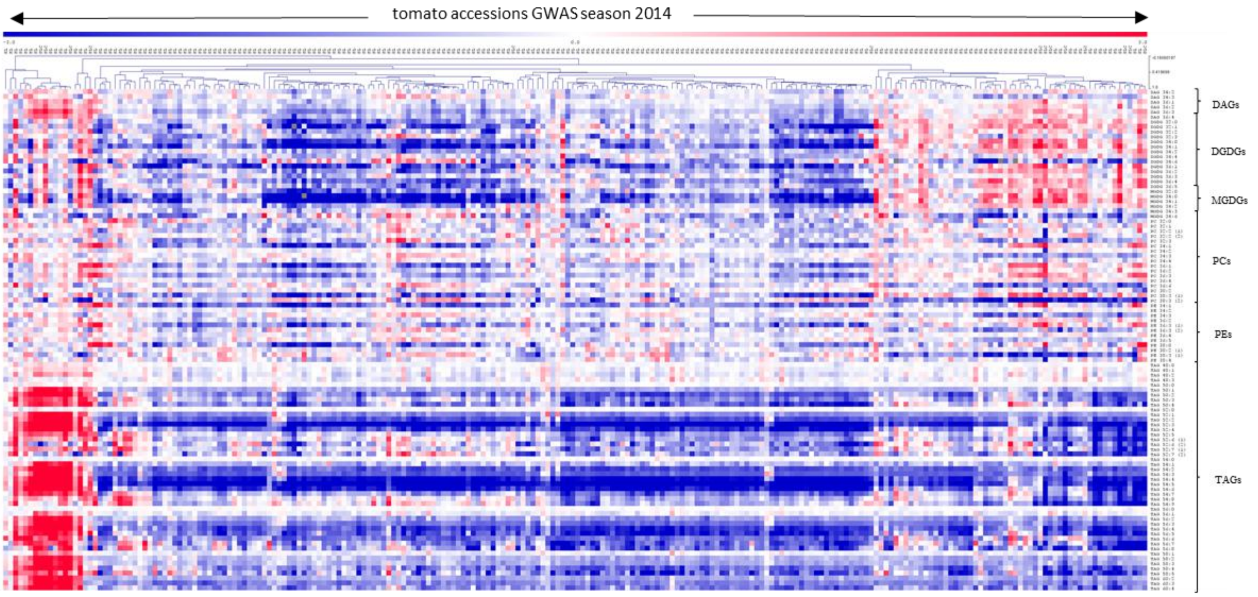

B

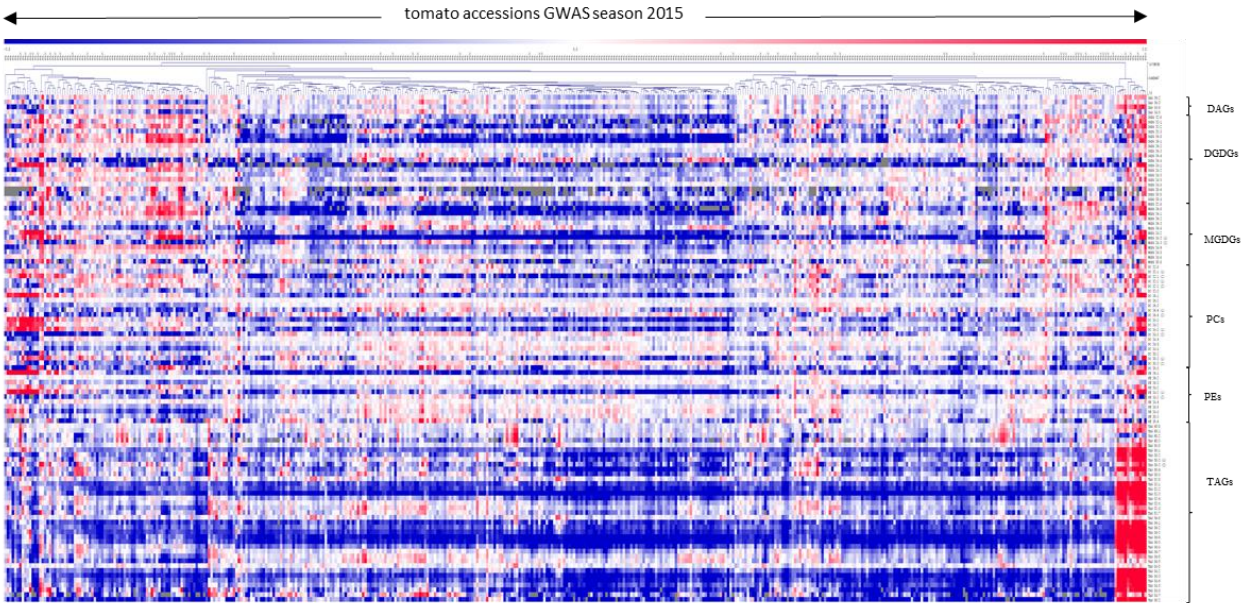

Figure S2. Heatmap of lipid levels across 550 accessions of the GWAS panel.
The data represent lipidomic profiling of material harvested in two consecutive years 2014 (A) and 2015 (B) of plants grown in the greenhouse. For each lipid species mean lipid level was calculated and the level of the same lipid in each accession was normalized to this mean by dividing each lipid value by this mean. Each season was normalized separately and presented in a logarithmic scale (log2). Regions of red or blue indicate lower or higher compared to the average of each lipid species, respectively.

Figure S3

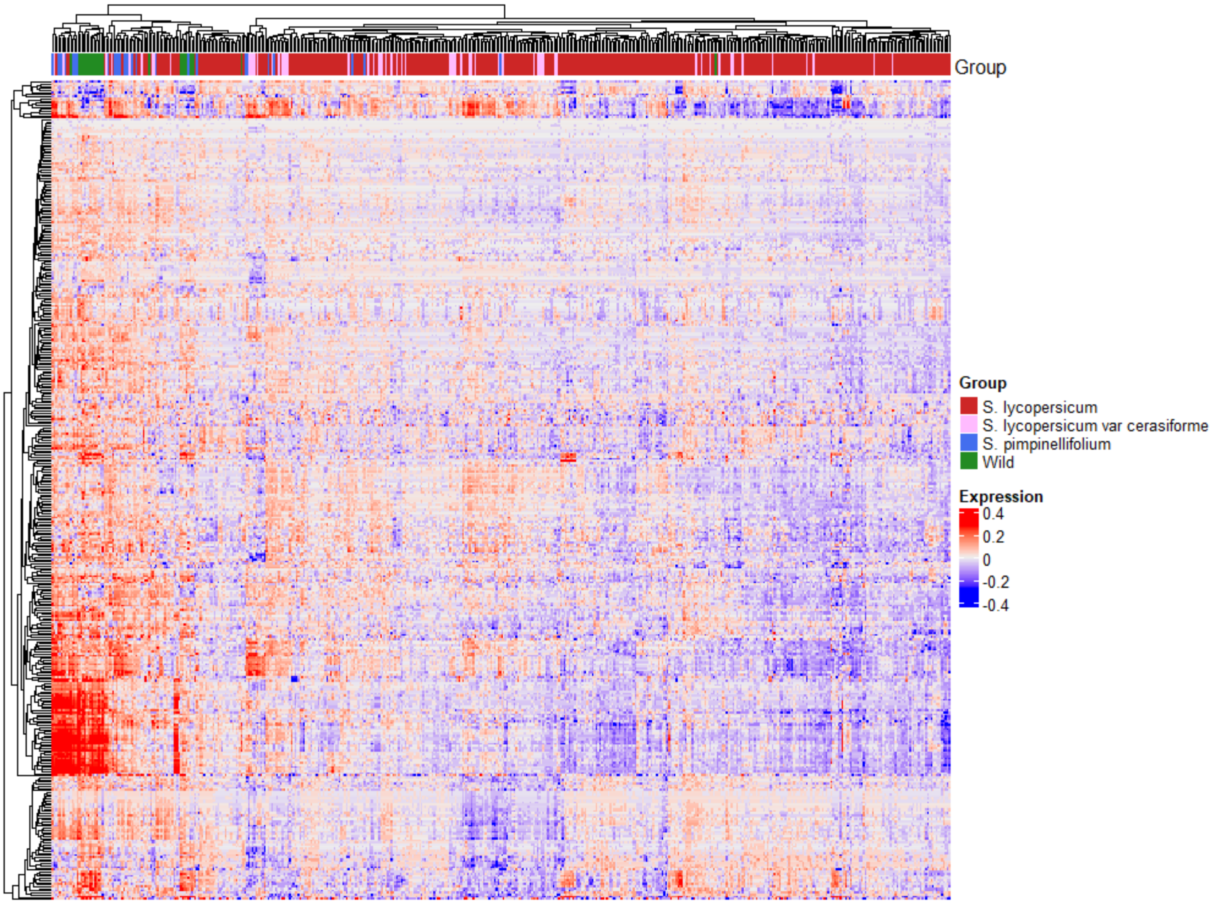

Figure S3. Heatmap of lipid levels across 550 accessions of the GWAS panel belonging to *S. lycopersicum*, *S. lycopersicum* var. *cerasiforme*, *S. pimpinellifolium*, and wild tomato groups.

For each lipid species mean lipid level was calculated and the level of the same lipid in each accession was normalized to this mean by dividing each lipid value by this mean. The data are presented in logarithmic scale (log2). Regions of red or blue indicate lower or higher compared to the average of each lipid species, respectively.

Figure S4

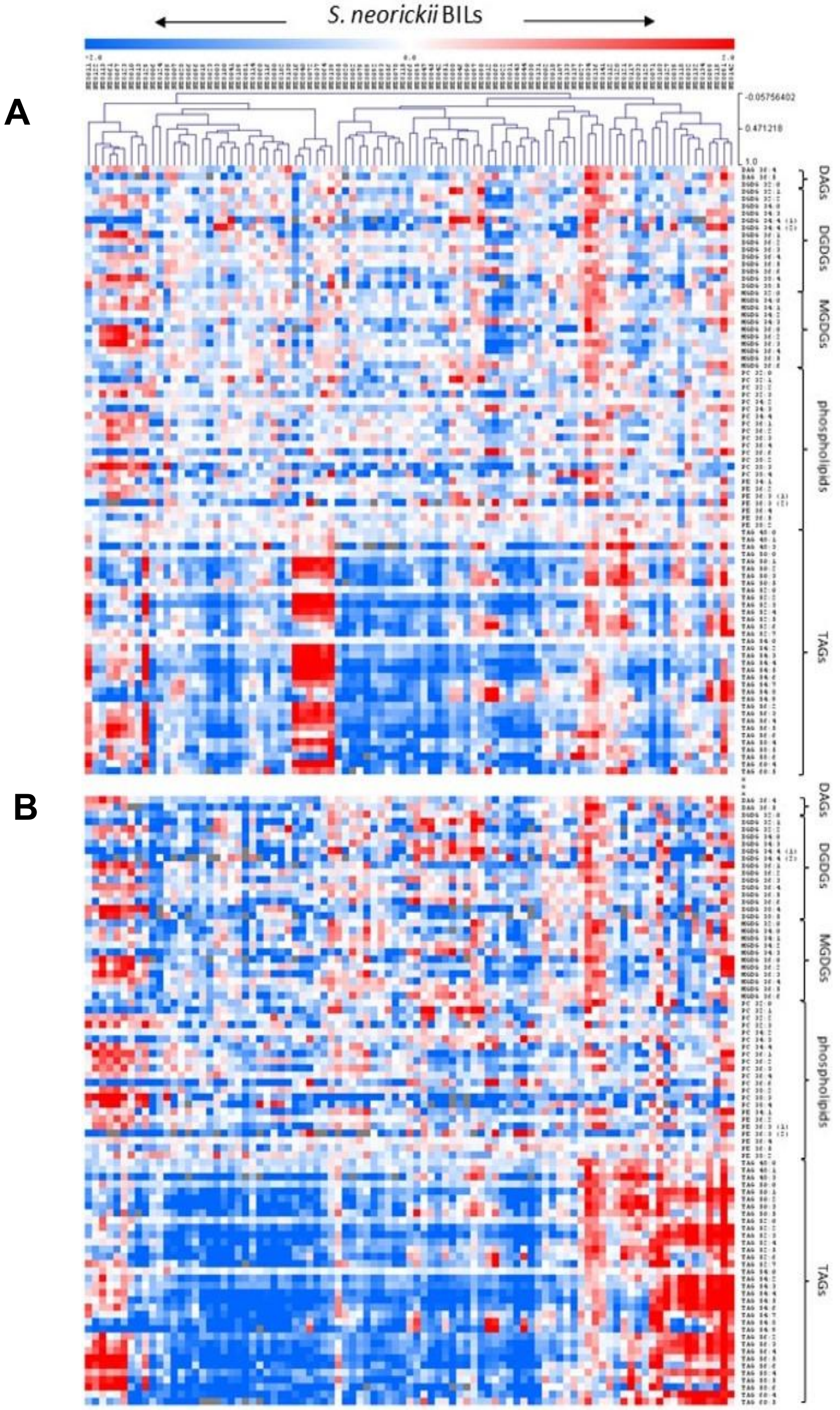

Figure S4. Heat map of lipid profiling across *S. neorickii* backcross inbred lines (BILs).

The data represent lipidomic profiling of material harvested from *S. neorickii* BILs population. (A) heterozygous and (B) homozygous lines. For each lipid species mean lipid level were calculated and the level of the same lipid in each BIL were normalized to this mean by dividing each lipid value by this mean. Each season was normalized separately and presented in a logarithmic scale (log2). Regions of red or blue indicate lower or higher compared to the average of each lipid species, respectively. Regions of white color, reflecting many of the chromosomal segment substitutions, do not affect lipid levels.

Figure S5

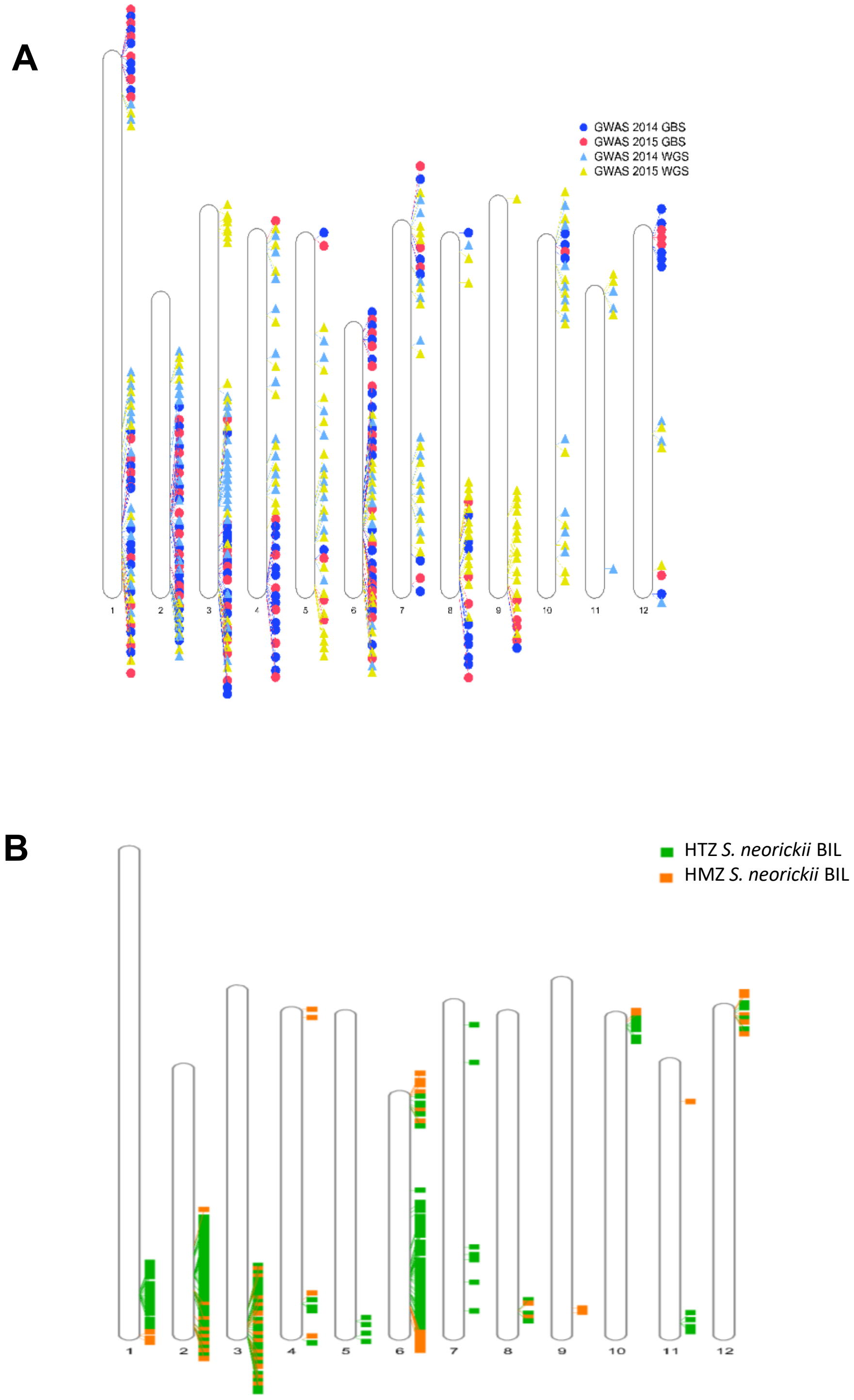

Figure S5. Chromosomal distribution of identified mQTL.
(A) Idiogram represents a chromosomal distribution of the mQTL resulting from GWAS of material harvested in two consecutive years using GBS and WGS SNPs data (B) Chromosomal distribution of the mQTL found in the BIL mapping of heterozygous and homozygous lines.

Figure S6

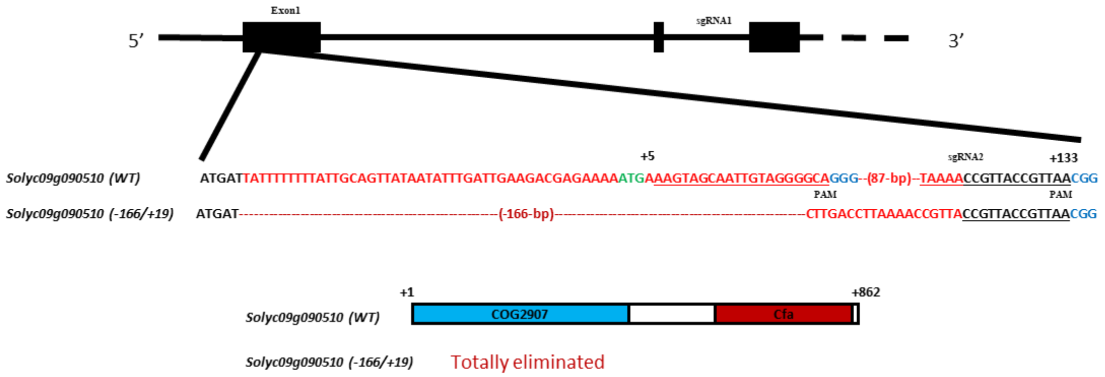

Figure S6. Construction and characterization of *CFAPS1*-edited lines.
CFAPS1 KO line (Fla. 8059 background) exhibits a deletion of 166 bp and an insertion of 19 bp in the first exon.

Figure S7

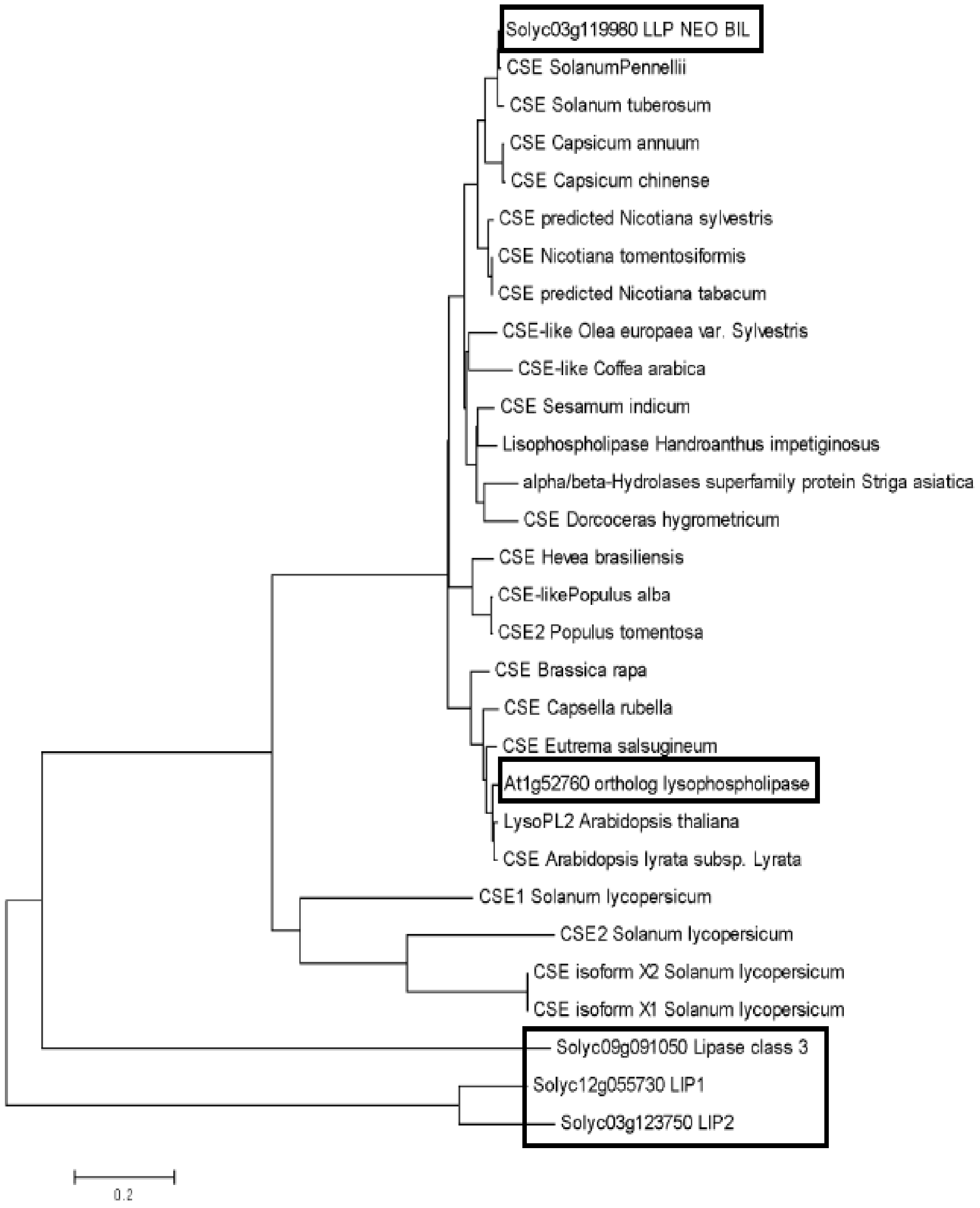

Figure S7. Phylogenetic analysis of the caffeoyl shikimate esterase (CSE) family. Coding sequences of genes with confirmed function as CSE or putatively annotated as CSE were used for the construction of a phylogenetic tree. Frame highlights two genes, the tomato *TomLLP* and *CSE* from Arabidopsis. Genes IDs are specified in Dataset S9. A phylogenetic tree was reconstructed with the neighbor-joining method.

Figure S8

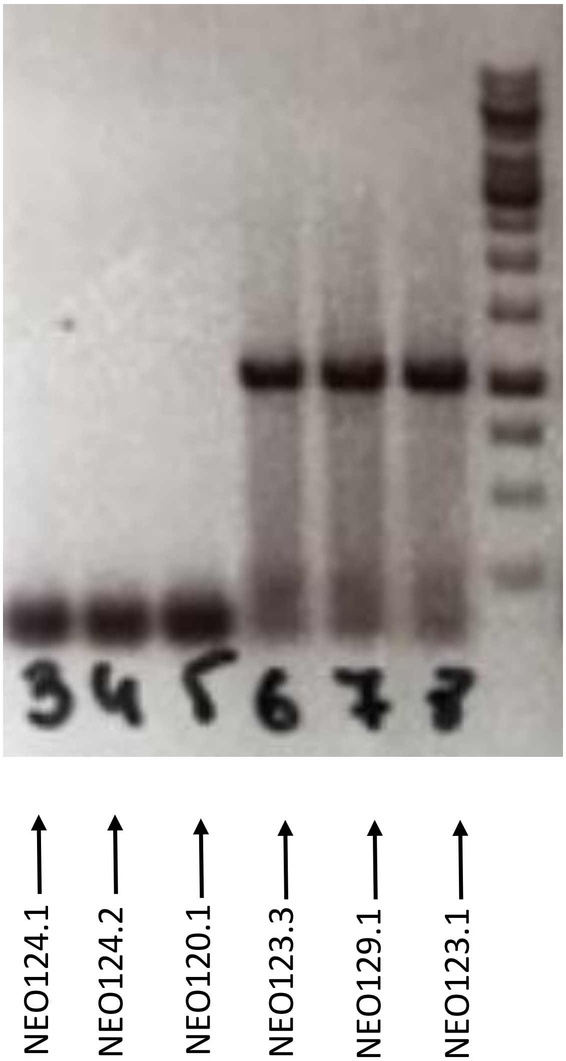

Figure S8. Expression level of *TomLLP* across six *S. neorickii* BILs. Monitoring the expression level of *TomLLP* using PCR on six BILs. Of which, in the region containing *TomLLP:* three BILs with the TA209 background (NEO124.1; NEO124.2; NEO120.1), and three BILs with the *S. neorickii* background (NEO123.3; NEO129.1; NEO123.1).

Figure S9

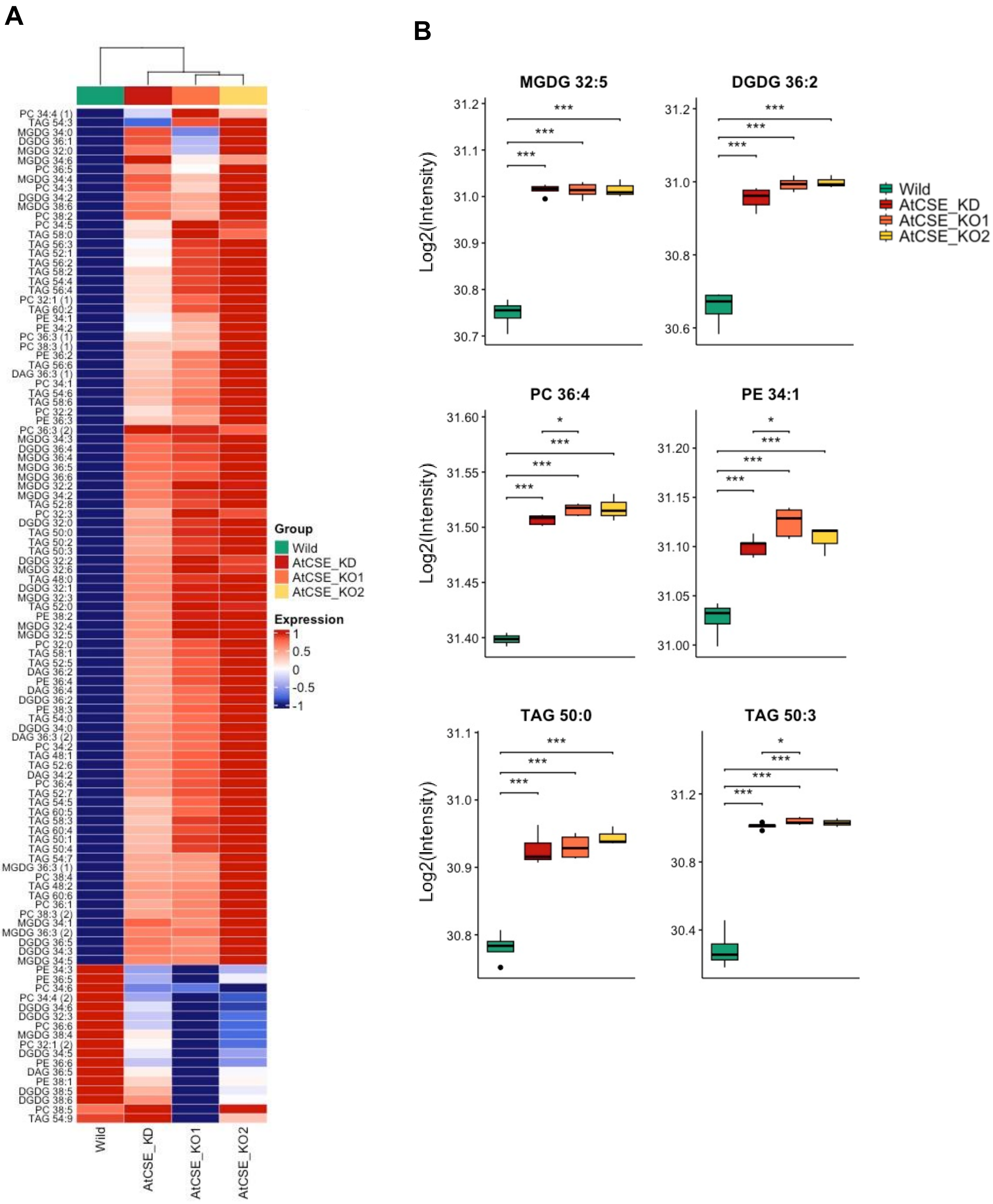

Figure S9. CSE (*At1g52760*) influences the lipid metabolism in Arabidopsis.
(A) Heatmap shows the significant ($p \leq 0.05$) changes in lipid levels between wild-type and the *cse* knock-out (KO) and knock-down (KD) lines. (B) Changes in lipid levels of selected lipids classes between the *cse* KO and KD lines and the wild type.

Figure S10

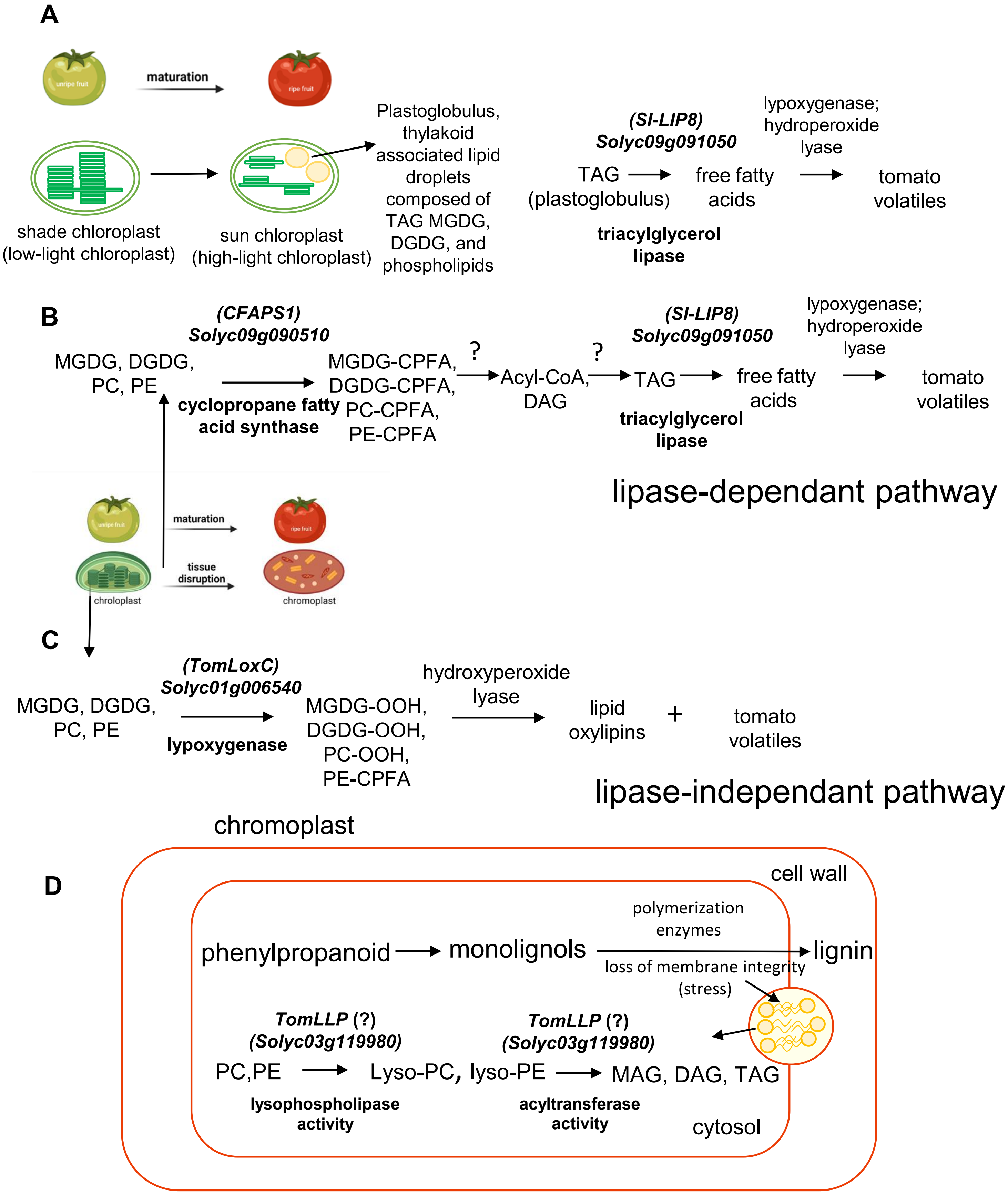

Figure S10. Schematic representation of biochemical pathways where identified candidate genes are potentially involved. (A) Schematic representation of the pathway for volatile synthesis from free fatty acids liberated from triacylglycerol associated with thylakoid membranes (plastoglobuli) by class III lipase (*Solyc09g091050*) under high-light conditions. (B) Schematic representation of the process converting membrane lipids (phospho- and galactolipids) to acylglycerols via cyclopropane fatty-acyl phospholipid, leading to subsequent volatile production. (C) Schematic representation of the process of lipid oxylipin and volatile production through lipoxygenase enzymes (Solyc01g06540) in the lipase-independent pathway. (D) Estimated role of the lipase-like protein (*Solyc03g119980*, orthologue of *At1g52760*) in lipid rearrangements during the lignin biosynthetic pathway. *Solyc03g119980* exhibits acyltransferase and hydrolase activities in lipid metabolism and is also involved in lignin biosynthesis.

Figure S11

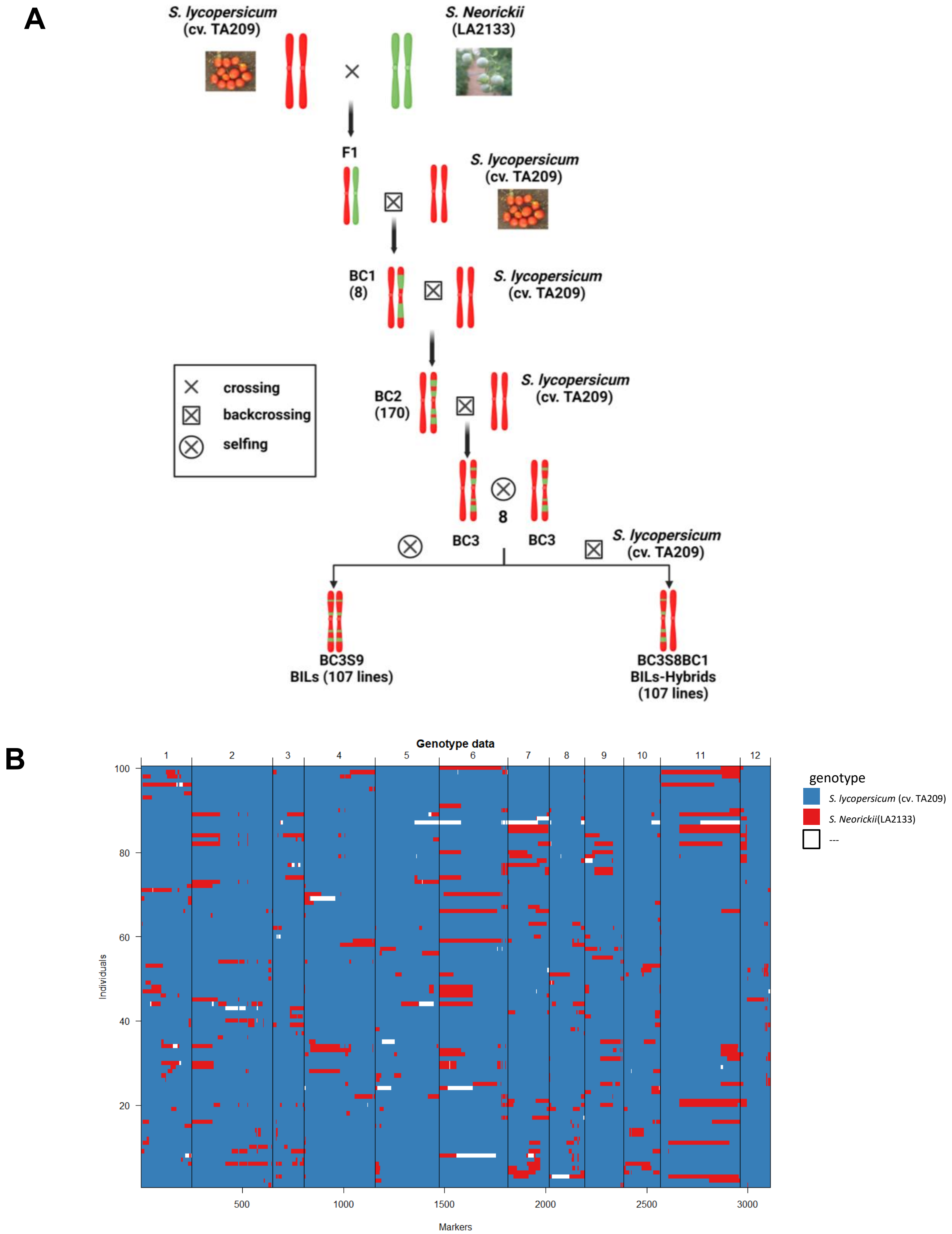

Figure S11. Breeding scheme and genetic map for backcross inbred lines (BILs).

(A) First cross: pollen from *S. neorickii* was placed onto the stigma of cv. TA209 to obtain F1 plants. Additional backcrosses with cv. TA209 was performed to decrease the *S. neorickii* genome introgression in the BILs. For each generation, the amount of plants is shown in parentheses. A final cross of the homozygous BILs with cv. TA209 was performed to obtain heterozygous lines (74). (B) Schematic representation of *S. neorickii* backcrossed inbred lines. The BILs harbor on average 4.3 introgressions per line, with a mean introgression length of 34.7 Mbp, allowing the division of the genome into 340 bins and enabling rapid trait mapping (74).
